# Immune Checkpoint Gene Expression Profiling Identifies Programmed Cell Death Ligand-1 Centered Immunologic Subtypes of Oral and Squamous Cell Carcinoma With Favorable Survival

**DOI:** 10.3389/fmed.2021.759605

**Published:** 2022-01-20

**Authors:** Yang Yu, Huiwen Tang, Debora Franceschi, Prabhakar Mujagond, Aneesha Acharya, Yupei Deng, Bernd Lethaus, Vuk Savkovic, Rüdiger Zimmerer, Dirk Ziebolz, Simin Li, Gerhard Schmalz

**Affiliations:** ^1^Department of Stomatology, Qunli Branch, The First Affiliated Hospital of Harbin Medical University, Harbin, China; ^2^Department of Experimental and Clinical Medicine, University of Florence, Florence, Italy; ^3^Regional Centre for Biotechnology, 3rd Milestone Gurgaon-Faridabad Expressway, Faridabad, India; ^4^Dr. D. Y. Patil Dental College and Hospital, Dr. D. Y. Patil Vidyapeeth, Pune, India; ^5^Laboratory of Molecular Cell Biology, China Tibetology Research Center, Beijing Tibetan Hospital, Beijing, China; ^6^Department of Cranio Maxillofacial Surgery, University Clinic Leipzig, Leipzig, Germany; ^7^Department of Cariology, Endodontology and Periodontology, University of Leipzig, Leipzig, Germany; ^8^Stomatological Hospital, Southern Medical University, Guangzhou, China

**Keywords:** PDL1, immune checkpoint genes, oral and squamous cell carcinoma, immune subtypes, prognosis

## Abstract

**Objective:**

This study aimed to identify the programmed death ligand-1 (PDL1, also termed as CD274) and its positively correlated immune checkpoint genes (ICGs) and to determine the immune subtypes of CD274-centered ICG combinations in oral and squamous cell carcinoma (OSCC).

**Materials and Methods:**

Firstly, the 95 ICGs obtained *via* literature reviews were identified in the Cancer Genome Atlas (TCGA) database in relation to OSCC, and such 88 ICG expression profiles were extracted. ICGs positively correlated with CD274 were utilized for subsequent analysis. The relationship between ICGs positively correlated with CD274 and immunotherapy biomarkers (tumor mutation burden (TMB), and adaptive immune resistance pathway genes) was investigated, and the relationships of these genes with OSCC clinical features were explored. The prognostic values of CD274 and its positively correlated ICGs and also their associated gene pairs were revealed using the survival analysis.

**Results:**

Eight ICGs, including CTLA4, ICOS, TNFRSF4, CD27, B- and T-lymphocyte attenuator (BTLA), ADORA2A, CD40LG, and CD28, were found to be positively correlated with CD274. Among the eight ICGs, seven ICGs (CTLA4, ICOS, TNFRSF4, CD27, BTLA, CD40LG, and CD28) were significantly negatively correlated with TMB. The majority of the adaptive immune resistance pathway genes were positively correlated with ICGs positively correlated with CD274. The survival analysis utilizing the TCGA-OSCC data showed that, although CD274 was not significantly associated with overall survival (OS), the majority of ICGs positively correlated with CD274 (BTLA, CD27, CTLA4, CD40LG, CD28, ICOS, and TNFRSF4) were significantly correlated with OS, whereby their low-expression predicted a favorable prognosis. The survival analysis based on the gene pair subtypes showed that the combination subtypes of CD274_low/BTLA_low, CD274_low/CD27_low, CD274_low/CTLA4_low, CD8A_high/BTLA_low, CD8A_high/CD27_low, and CD8A_high/CTLA4_low predicted favorable OS.

**Conclusion:**

The results in this study provide a theoretical basis for prognostic immune subtyping of OSCC and highlight the importance of developing future immunotherapeutic strategies for treating oral cancer.

## Introduction

Oral and squamous cell carcinoma (OSCC) is one of the most common oral cancers and is characterized by high morbidity and mortality (1). Currently applied treatment approaches include tumor resection, radiotherapy, and adjuvant chemotherapy but patients with OSCC continue to display an unsatisfactory prognosis after such routine therapy (2). The median survival period of patients with OSCC is 515 days, which is <1.5 years (3). This fact underscores a urgent need for innovative and effective therapeutic strategies for treating OSCC. Immunotherapy based on the drugs targeting immune checkpoint genes (ICGs) has gained considerable attention in recent years, with a surge in the development of novel immune strategies to treat and improve the survival of patients with cancer (4). Two main approaches have been proposed to enhance the antitumor immunity. The most well-investigated approach is the blockade of coinhibitory molecules with monoclonal Abs directed to T-cell surface ICG biomarkers, namely programmed cell death protein-1 (PD1)/programmed death ligand-1 (PDL1 also named as CD274), CTLA4, LAG3, TIM3, and B- and T-lymphocyte attenuator (BTLA) (5). The second approach is based on activating costimulatory molecules with agnostic Abs directed to T-cell surface ICG biomarkers, namely CD27, CD40, OX40, and CD137 (6). Most current research is focused on coinhibitory ICGs due to the fundamental safety challenges accompanying the triggering costimulatory immune pathways, as well as the dose-limiting toxicities of mAbs agents to costimulatory ICGs (7). Therefore, the primary focus of researchers has been directed at investigating coinhibitory ICGs, particularly PD1 and its ligand-PDL1 (8).

It is well-established that PDL1 expressed on the surface of cancer cells can bind with PD1 on the surface of T-cells, and the interaction between PDL1 and PDL1 can inhibit the function of T-cells by inhibiting proliferation, promoting apoptosis, and inhibiting the cytokine secretion (9). The inhibitor drugs targeting PDL1, pembrolizumab and pembrolizumab, have been approved for use in many solid cancer treatments, including the first-line treatment for patients with recurrent or metastatic head and neck squamous cell carcinomas (HNSCC) (10). However, currently, there are no clinical trials of PDL1 inhibitors for treating OSCC. Prior clinical trials using a PDL1 inhibitor in HNSCC showed unsatisfactory response rates in an unselected population of patients with cancer, suggesting that not all patients respond well to the PDL1 inhibitor-based immunotherapy and a subset of patients are resistant to such immunotherapy (11). The purported cause underlying this phenomenon is to vary the expression levels of PDL1 among patients; therefore, patients with a high expression of PDL1 might achieve clinical responses, while those with a low expression are likely to be resistant. Another important influencing factor might be the density, composition, and activation state of the CD8+ effector T-cells, which play a central role in antitumor immunity. Patients with low infiltration of CD8+ T-cells might not respond to such immunotherapy (12). Based on these factors, there is an urgent need for the differentiation of patients with cancer into immune subtypes according to the expression level of PDL1 and the infiltration of CD8+ T-cells. The subtyping of an immune microenvironment in OSCC can be beneficial for identifying patients who may benefit from ICG-targeting therapies, and aid in optimizing the agent design for future clinical trials.

Furthermore, a few studies have shown that coinhibitory ICGs' inhibitor drugs, when administered as synergistic combination strategies, induced a dramatic increase in durable response rates as compared to monotherapy (13). The combination of PDL1 and its synergistic ICG blockers was shown to significantly increase the response rates and enhance the treatment efficacy in patients with cancer. For example, the combination of ipilimumab (anti-CTLA-4) plus nivolumab (anti-PD-1) was shown to significantly enhance efficacy in patients with metastatic melanoma; therefore, ipilimumab plus nivolumab was approved for the treatment of metastatic melanoma, advanced renal cell carcinoma, and metastatic colorectal cancer (14). The treatment efficacy of PDL1-centered ICG combination inhibitors has been reported in many research studies on melanoma, breast cancer, and lung cancer (15, 16). The success of this combination in other cancer types encourages its investigation in OSCC. While previous studies have addressed the synergistic combination of coinhibitory ICG inhibitors in cancer treatment, it has not yet been investigated in the context of OSCC.

To address this research gap, the present study utilized the Cancer Genome Atlas (TCGA) (17) and the Gene Expression Omnibus (GEO) database (18). The TCGA is well-established as the most comprehensive cancer genomics program, which has produced, evaluated, and made public data related to the genomic sequencing, expression, methylation, and copy number variation of over 11,000 patients with cancer who have been diagnosed with more than 30 distinct forms of cancer (17). Many previous bioinformatics studies have followed the traditional study design of analyzing the TCGA data and followed by the verification of the computationally predicted results using GEO data sets (19–24). The GEO database is a freely accessible resource for the functional genomics data that contain original data sets from tens of thousands of published microarray or sequencing experiments (25). Because GEO data sets vary in the aspects of experimental design, country, race, laboratories, experimental platform, sample size, and disease severity, these multiple factors allow the GEO data sets to be heterogeneous. If the results determined based on the TCGA data can be validated using the heterogeneous GEO data sets, the predicted results can be considered reliable. Therefore, in the present investigation, four oral cancer GEO data sets were used as independent cohorts to validate the prognostic immune subtypes results obtained using the TCGA data.

Based on the clinical survival data and RNA expression data from the TCGA and GEO database, the present research aimed to identify the ICGs that work synergistically with PDL1 in OSCC, as well as the PDL1-centered ICGs combinations that have prognostic values for OSCC. The ICG inhibitor combinations and prognostic immune subtypes identified in this research could provide novel strategies for the immunotherapy of OSCC, and bear the potential for clinical translation.

## Materials and Methods

### Study Design

**Figure 1:**
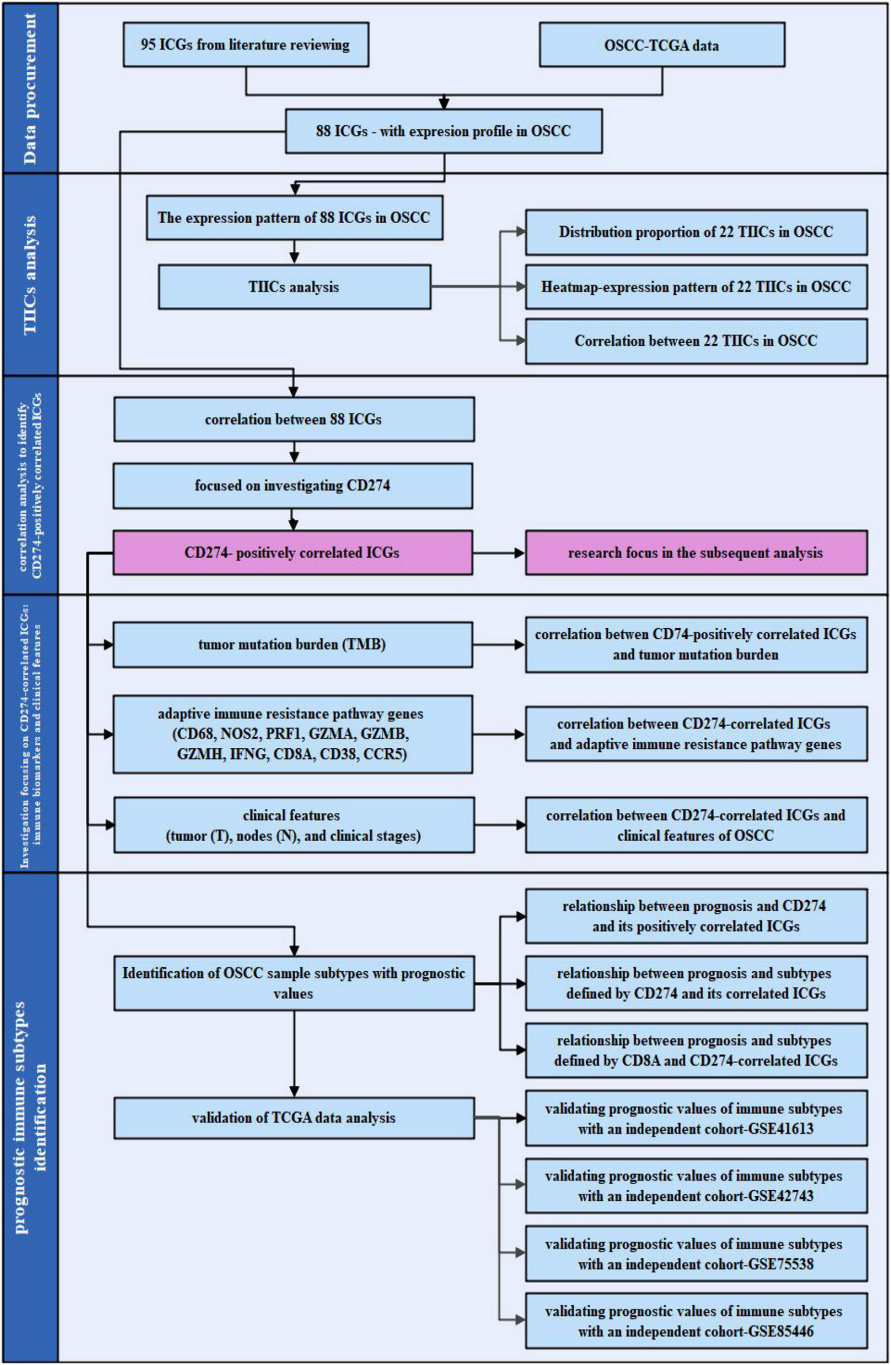
The work flowchart of the present study. The flowchart is divided into four steps: data procurement for obtaining the 88 immune checkpoint genes (ICGs) with the expression profile in the Cancer Genome Atlas (TCGA) oral and squamous cell carcinoma (OSCC) data set; tumor-infiltrating immune cells (TIICs) analysis; a correlation analysis for identifying ICGs positively correlated with CD274; and subsequent analysis focusing on the ICGs positively correlated with CD274 particularly from the aspect of the tumor mutation burden (TMB), adaptive immune resistance pathway, and clinical features; as well as the identification of the prognostic immune subtypes.

The work flowchart is shown in Figure 1. In brief, 95 ICGs obtained from the literature review were mapped into the OSCC-TCGA data set, and 88 ICGs were found in the data set. The tumor-infiltrating immune cell (TIIC) analysis was performed based on the expression profiles of 88 ICGs in OSCC samples, and it included distribution proportion, a heat map analysis, and a correlation analysis. The correlation among the 88 ICGs in OSCC samples was investigated, particularly focusing on the ICGs positively correlated with CD274. Afterward, the relationship between ICGs positively correlated with CD274 and several immunotherapy-related aspects was investigated. Moreover, the OSCC sample subtypes with prognostic values were identified by investigating the prognostic values of the sole genes (CD274 and its positively correlated ICGs); the prognostic values of the combination subtypes defined by CD274 and its positively correlated ICGs; and the prognostic values of the combination subtypes defined by ICGs positively correlated with CD8A and CD274. To validate the prediction accuracy of the prognostic immune subtypes identified by the TCGA data analysis, four independent cohort data sets (i.e., GSE41613, GSE42743, GSE75538, and GSE85446) were used for verification.

### Procurement of 95 ICGs by Literature Reviews

The ICG list, including 95 ICGs, was obtained by collecting the union of ICG list in several literatures investigating the involvement of ICGs in cancers (26–29). ICGs are displayed in Supplementary Table S1.

### OSCC Data Downloading and Preprocessing

The HNSCC data from the TCGA database were downloaded from the University of California Santa Cruz's official webpage (https://xenabrowser.net/datapages/). The downloaded data regarding HNSCC consisted of gene expression RNAseq data, survival data, somatic mutation (SNPs and small INDELs) data, curated clinical data, and phenotype data. The OSCC data were collected by selecting the OSCC-related anatomic sites in the column of “site_of_resection_or_biopsy.diagnoses” of the excel file regarding the clinical data of HNSCC. The OSCC-related anatomic sites include buccal mucosa, retromolar trigone, alveolar ridge, the floor of the mouth, hard palate, oral cavity, gingiva (upper and lower), and oral tongue (anterior 2/3) (30). Afterward, the OSCC data were pre-processed by removing the samples without clinical information, particularly survival information, and the samples with the overall survival (OS) of <1 month, and the adjacent healthy control samples, as well as the samples in which genes' Fragments Per Kilobase of transcript per Million mapped reads (FPKM) value was 0. After performing such preprocessing, the 326 OSCC samples were obtained. Supplementary Table S2 presents the sample size belonging to the different anatomic sites of the 326 OSCC samples. As seen from Supplementary Table S2, the most frequent site is the tongue (42.33%); the overlapping lesion of the lip as the second frequent site (25.15%); and the floor of the mouth as the third frequent site (16.87%).

### Procurement of the Expression Profiles of 88 ICGs in OSCC Samples

The ensemble IDs of genes in the expression profile of OSCC were converted to the gene SYMBOL by using the Bioconductor package (31). Regarding the genes that repeatedly appear after conversion, the average expression values of these genes were taken to ensure that the genes were unique in the expression profile. Afterward, 95 ICGs were mapped into the expression profile of OSCC, and 88 ICGs showed an expression in the TCGA-OSCC data set, and thus the expression profile of these 88 ICGs was obtained.

### Analysis of TIICs in OSCC Samples

Twenty-two TIICs were obtained based on the CIBERSORT webtool (https://cibersort.stanford.edu/) (32). Firstly, the expression profiles of 88 ICGs in TCGA-OSCC samples were normalized, and the proportion of TIICs in OSCC samples was predicted using the CIBERSORT webtool (32). A total of 115 OSCC samples were obtained from 326 TCGA-OSCC samples by selecting the value of *p* < 0.05. Secondly, the expression levels of varying ICGs in each type of cell were obtained. The average value of all ICGs in a certain type of cell was regarded as the expression level of this type of cell in samples. The heat map was plotted to show the expression levels of 22 TIICs in 115 OSCC samples.

Thirdly, a correlation plot was drawn based on the expression levels of TIICs in 115 samples to analyze the correlation between TIICs in the pathogenesis of OSCC. The Pearson correlation coefficient was used for calculating the correlation between any two types of TIICs. The correlation relationship between the two ICGs is represented by the letter *r* and quantified with a number, which varies between −1 and +1. Zero means that there is no correlation, whereas 1 means a complete or perfect correlation. The sign of *r* shows the direction of a correlation: a positive *r* means that the certain two ICGs were positively correlated and can play a synergistic role and vice versa. The interpretation of the Pearson's correlation coefficients value should be referred to the literature by the users' guide provided by Haldun Akoglu in 2018 (33). The |*r*| value ≥ 0.8 indicates a very strong correlation; 0.5 ≤ |*r*| < 0.8 indicates a moderate correlation; 0.3 ≤ |*r*| < 0.5 indicates a fair correlation; and |*r*| < 0.3 indicates a poor correlation.

### Heat Map Shows the Prognostic Values of 88 ICGs in OSCC

Eighty-eight ICGs with expression values in the TCGA-OSCC data set were obtained. Differentially expressed ICGs in the OSCC-TCGA data were identified by performing a differential expression analysis and using the edgeR package (version 3.14) (34) in the R software (version 3.6.3). Genes with log FC > 0 and the value of *p* < 0.05 were regarded as upregulated differentially expressed genes (DEGs); while genes with logFC < 0 and the value of *p* < 0.05 were regarded as downregulated DEGs (35). The relationship between these 88 ICGs and the OS of patients with OSCC was analyzed using a univariate Cox regression analysis (log-rank *p* < 0.05). Heat maps were plotted using the pheatmap package (version 1.0.12) in R (36).

### The Correlation Relationship Among 88 ICGs

To evaluate the correlation relationship among 88 ICGs, the Spearman algorithm was used to calculate the correlation of the expression value of 88 ICGs. The correlation relationship among 88 ICGs was displayed by using the corrplot package (version 0.92) in R (37). The correlation between PDL1 (CD274) and other ICGs was particularly marked as the research focus in subsequent analysis aimed to identify the ICGs, which were either positively (synergistic) or negatively (antagonistic) correlated with CD274.

### Identification of Prognostic ICGs Positively Correlated With CD274

Among the 88 ICGs, ICGs, which were positively correlated to CD274 and had the value of *p* < 0.05, were selected. Based on the expression values of these selected genes, the univariate Cox regression analysis was performed by using the survival package (version 3.2-13) in R (38). The relationship between the expression values of selected ICGs and prognosis was shown by the forest plot, which was plotted by using the forestplot package (version 2.0.1) in R (39). ICGs, which were not only positively correlated with CD274 but also had significant prognostic values, were identified and used as the investigation focus in subsequent analysis. Furthermore, the functional enrichment analysis was performed to determine the significant biological processes (BPs), and signaling pathways enriched by the ICGs were identified in the last step. The gene names of these ICGs were converted to the Entrez ID using the org.Hs.eg.db package (version 3.14) in R (40). The functional enrichment analysis was performed using the clusterProfiler package (version 3.14) in R (41). The species for the analysis was selected to be Homo sapiens. The GO terms, particularly BP and KEGG pathways that were significantly enriched by the correlated genes, were identified by setting the threshold value of *p* < 0.05. If there are more than 20 terms that were significantly enriched by this threshold setting, then only the top 20 terms ranked by the ascending order of the value of *p* were obtained to plot the bubble chart; otherwise, if there are <20 terms that were significantly enriched by this threshold setting, then all of the terms were used for plotting the bubble chart. The bubble charts were plotted to visualize the enrichment results using the ggplot2 package (version 3.3.5) in R (42).

### Relationship Between TMB and CD274-Related ICG Group

Among the 326 OSCC samples obtained by data preprocessing, 320 samples appeared to be with somatic mutation. The tumor mutation burden (TMB) values of these 320 samples were first calculated. The expression profile of CD274 and its positively correlated ICGs in these 320 samples were obtained. Based on the TMB values of 320 samples and the expression profile of the CD274-related ICGs group, the correlation analysis was performed to show the relationship between TMB and CD274-related ICGs group using the Spearman correlation method (43). The value of *p* showing a correlation was displayed with the radar chart using the fmsb package (version 0.7.2) in R (44). In addition, Spearman's RHO correlation values were calculated, and scatterplots were created to assess the correlation between CD274-related ICGs group and TMB in 320 samples. The scatter plots were drawn by using the ggplot2 package (version 3.3.5) in R (42). The edge density maps, which were located on both sides of the scatter plot, were drawn by using the ggMarginal function of the ggExtra package (version 0.9) in R (45). The correlation coefficient and the values of *p* were added to the scatter plots by using the stat_cor function of the ggpubr package (version 0.4.0) in R (46).

### The Correlation Relationship Between Genes Involved in Adaptive Immune Resistance Pathway and Genes in the CD274-Related ICG Group

CD8+ T-cells can produce IFNγ and thus activate the immune pathways, leading to the upregulation of genes involved in an adaptive immune resistance pathway (e.g., CD68, NOS2, PRF1, GZMA, GZMB, GZMH, IFNG, CD8A, CD38, and CCR5). The correlation relationship between the genes involved in an adaptive immune resistance pathway and the genes in the CD274-related ICGs group was calculated by performing the Spearman correlation analysis based on the cor.test function in R. The Spearman correlation values and the values of *p* were obtained, based on which a heat map was plotted.

### Relationship Between ICGs and Their Clinical Features

Based on the clinical information obtained from the TCGA database, the relationship between the CD274-correlated ICGs and their clinical features was analyzed, particularly focusing on the tumor (T), node (N), and clinical stages. Box plots were drawn by using the ggboxplot function in the ggpubr package of R (46). The significance of test was performed by using the stat_compare_means function in the ggpubr package of R (46). The Kruskal–Wallis algorithm was used to examine the values of *p*, showing the relationship between the gene expression level and its clinical features.

### Relationship Between Prognosis and Subtypes Defined by CD274-Related ICG Group

The survival analysis was performed in three steps: (i) Firstly, to identify the relationship between the sole gene (CD274 and its positively correlated ICGs) and 5-year OS rate; (ii) Secondly, to identify the prognostic values of the combination of the genes consisted of CD274 and its positively correlated ICGs; (iii) Thirdly, to identify the prognostic values of the combination of the genes consisted of ICGs positively correlated with CD8A and CD274.

The survival analysis was performed by using the survival package in R (38). Kaplan–Meier (KM) plots were generated using the survfit function (version 3.2-13) in R (47); meanwhile, the values of *p* from log-rank tests were calculated. The clinical OSCC samples with the survival time of <5 years were obtained. By using the coxph function of the survival package in R, a Cox risk model was constructed for each gene (48). After then, the predict function was used for predicting the risk score of each model, and further the risk score of each sample was obtained. By taking the median value of the risk scores, the samples were divided into a high-expression group (H) and a low-expression group (L).

Regarding the second step of the survival analysis, the high-/low-expression groups of CD274 and its positively correlated ICGs were integrated. OSCC samples were divided into four categories to analyze the survival of gene classification samples.

Regarding the third step of the survival analysis, CD8A was selected among the adaptive immune resistance pathway genes, and it was focused in this section. The subtypes constituted by CD8A and each CD274-related ICG group gene were analyzed. Likewise, OSCC samples were divided into four categories to analyze the survival of gene classification samples.

### Validating the Prognostic Values of the Immune Subtypes Identified by TCGA Data

To validate the prognostic values of the immune subtypes identified by the TCGA data, four OSCC-related GEO data sets [i.e., GSE41613 (49), GSE42743 (49), GSE75538 (50), and GSE85446] with the OS information were obtained, based on which the survival analysis was performed. The survival data of patients with OSCC within 5 years were obtained for an analysis. Firstly, the relationship between ICGs positively correlated with CD274 and OS within 5 years was validated. Secondly, the prognostic values of gene pair subtypes consisting of ICGs positively correlated with CD8A and CD274 were validated.

## Results

### TIICs in OSCC Samples

**Figure 2:**
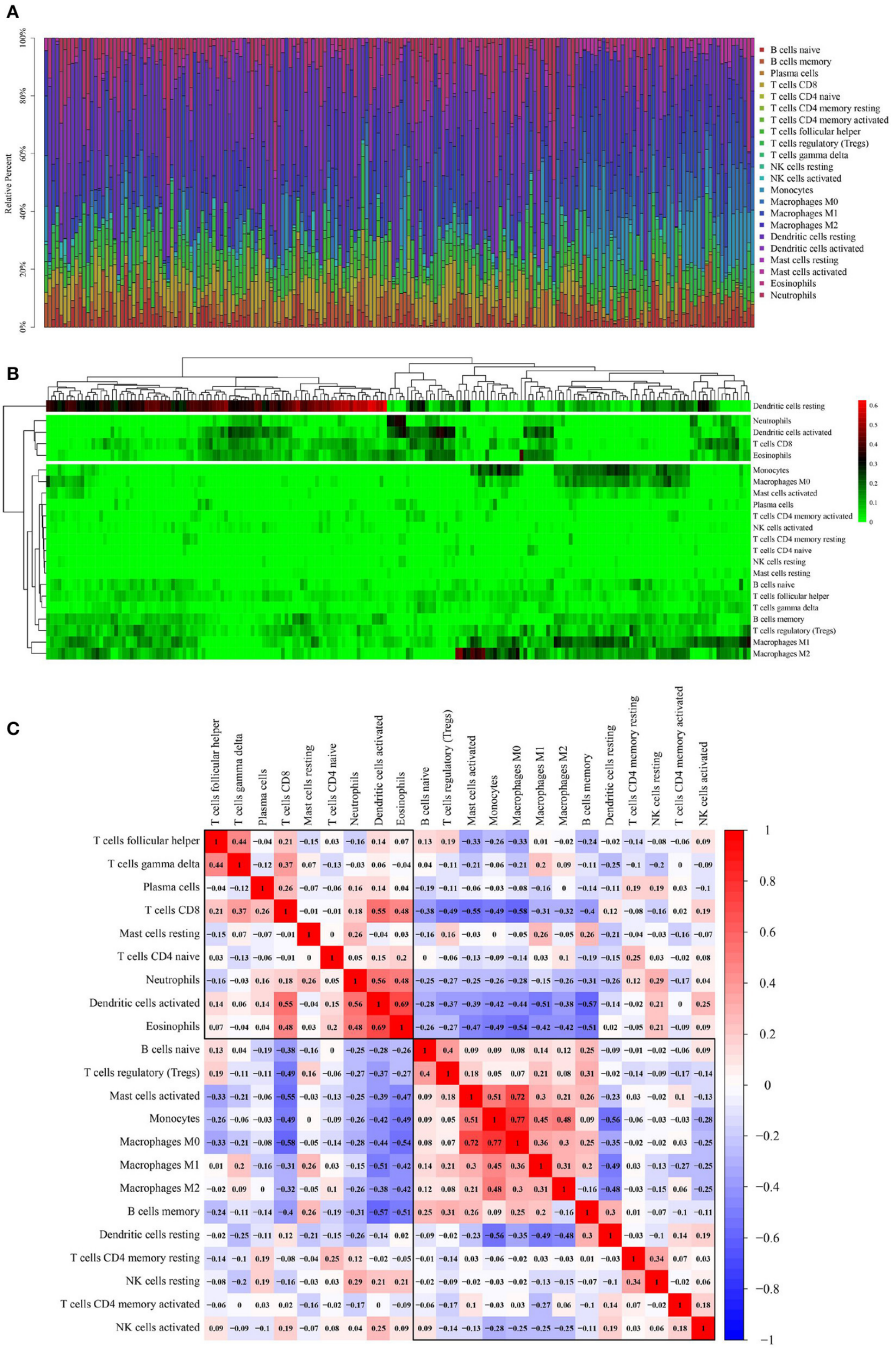
Performance of CIBERSORT ascertained TIICs in OSCC. **(A)** The distribution of 22 TIICs in OSCC samples. X-axis represents the name of 115 samples, and y-axis represents the composition ratio of the cells in each sample. Different colors represent different types of cells. The longer column of each cell in a certain sample indicates that the proportion of this type of cell is higher in this sample. **(B)** A heat map of the 22 TIICs proportions in 115 OSCC samples. Each column represents a sample, and each row represents one type of immune cell population. The levels of the immune cell populations are shown in different colors, which transition from green to red with increasing proportions. Y-axis represents the expression levels of TIICs in each sample. In the color bar, green represents a low expression of TIICs in samples; red represents a high expression of TIICs in samples; and black represents that the TIICs were not expressed in the samples, meaning the expression level was 0. **(C)** A correlation matrix of 22 immune cell proportions and immune/stromal score in OSCC. Variables have been ordered by average linkage clustering. For comparison, the immune/stromal score has been rescaled to range between 0 and 1 separately in each study. The correlation between TIICs in the pathogenesis of OSCC. Both the x- and y-axis represent the 22 types of TIICs. The color bar shows the correlation value of TIICs. Blue indicated that the TIICs were negatively correlated, and red indicated that the TIICs were positively correlated. The darker color indicated that the correlation showed a higher significance. The diagonal line drawn from the coordinate (0,22) to the coordinate (22,0) has a correlation of 1.

A total of 115 OSCC samples were obtained by selecting samples with the value of *p* < 0.05 from the CIBERSORT webtool. The proportion of 22 TIICs in each sample is shown in Figure 2A. A heat map shows the expression levels of 22 TIICs in 115 OSCC samples (Figure 2B). As observed from Figure 2B, dendritic cells who were at rest were highly expressed in OSCC samples, and the other types of cells were downregulated or nearly nonexpressed in OSCC samples.

Figure 2C shows the correlation among TIICs in the pathogenesis of OSCC. Because ICGs act as an interaction between tumor cells and T-cells, the interpretation of the results of this correlation analysis should also be focused on T-cells, particularly CD8+ effector T-cells and regulatory T- (Treg-) cells. Thereby, CD8+T-cells were significantly negatively correlated with macrophage M0 (Pearson correlation value = −0.58) and significantly positively correlated with activated dendritic cells (Pearson correlation value = 0.55); Treg cells were significantly negatively correlated with CD8+ T-cells (Pearson correlation value = −0.49).

### The Expression Pattern and Correlation Relationship of ICGs in OSCC Samples

**Figure 3:**
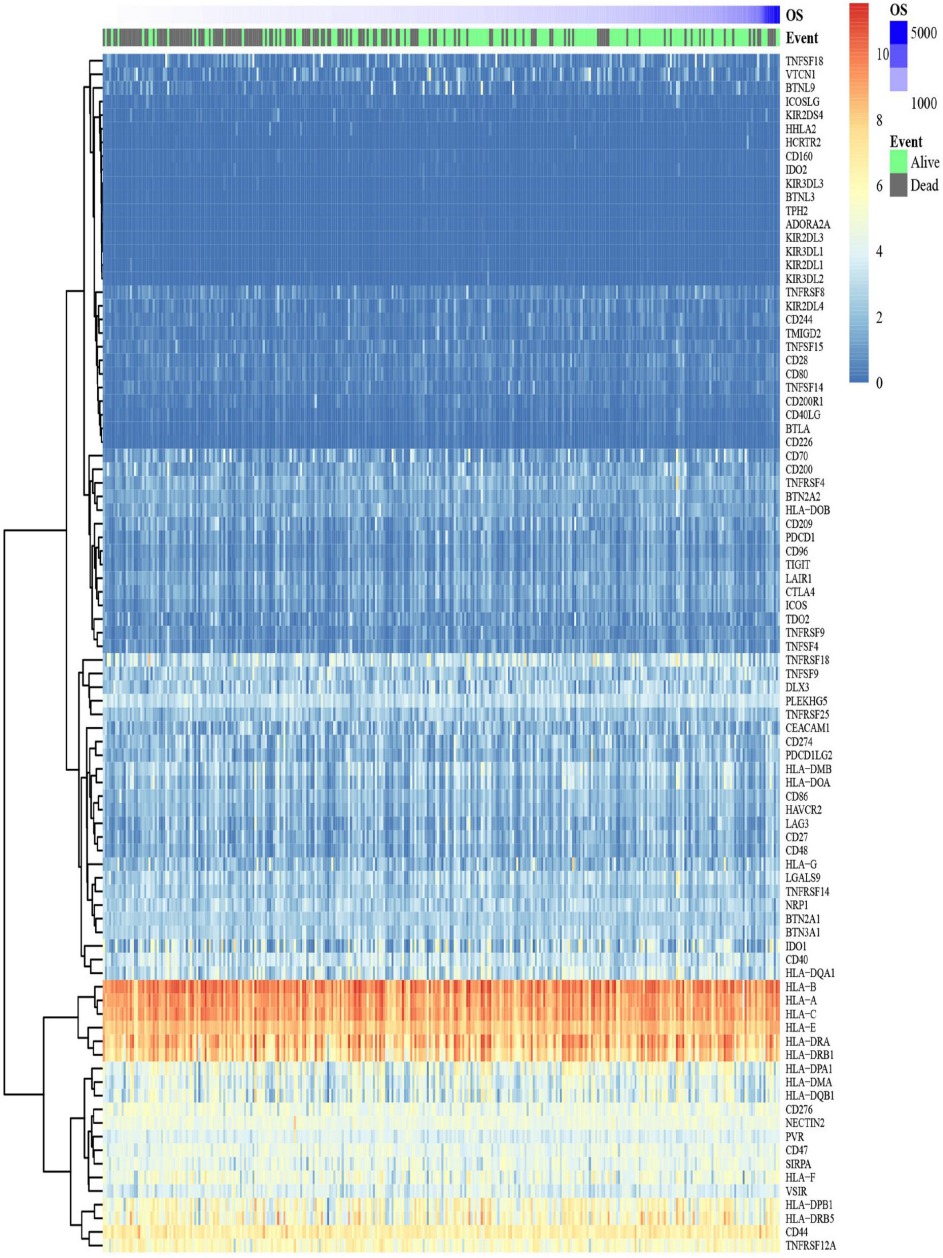
A heat map shows the expression pattern of ICGs in the TCGA-OSCC data set. The color bar refers to the gene expression levels. Red indicates relatively higher gene expression levels, and blue indicates relatively low gene expression levels.

**Figure 4:**
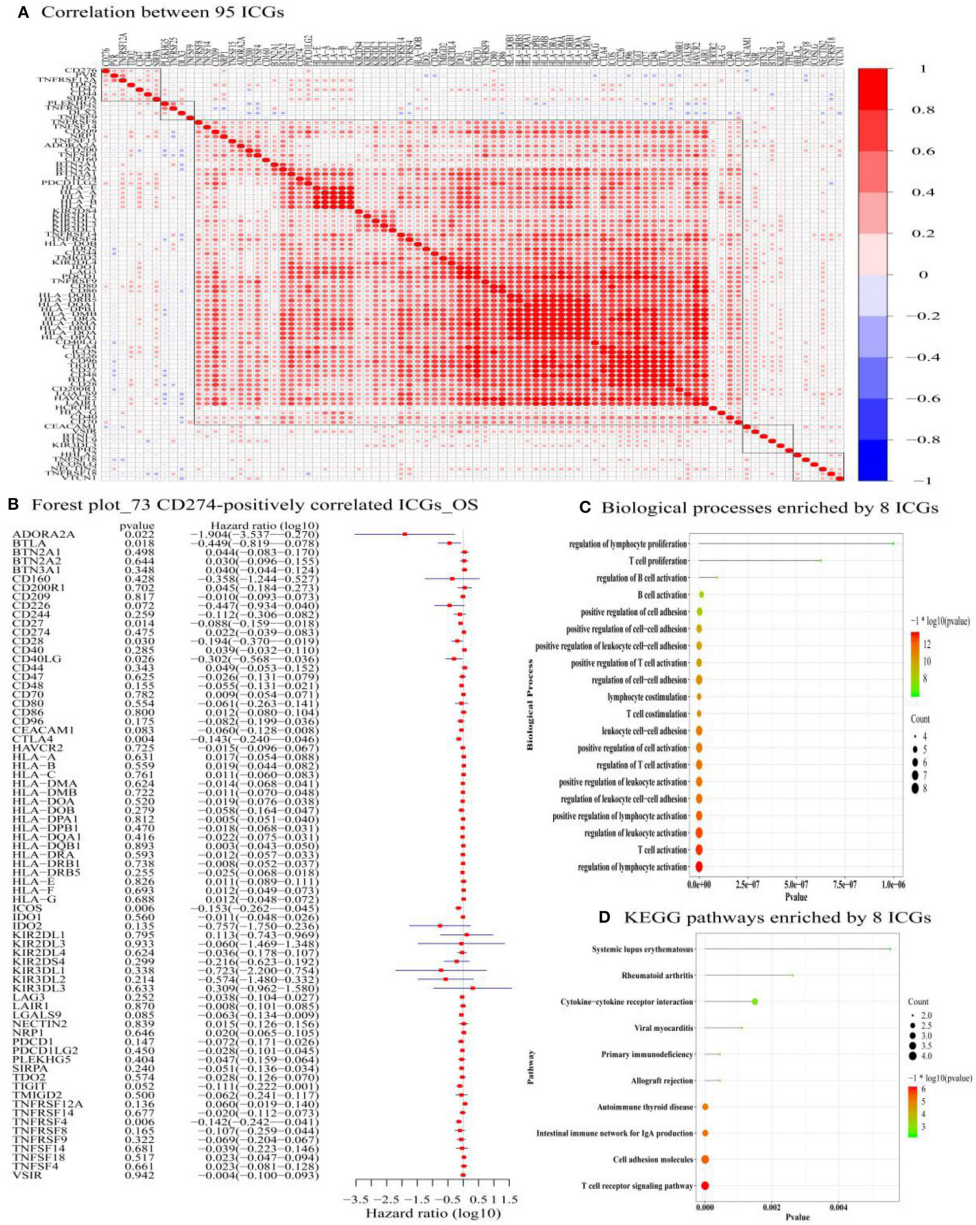
The correlation between 95 ICGs and the prognostic values of 73 CD274-correalted ICGs. **(A)** The correlation between 95 ICGs. The index of the color bar indicates that the positively correlated ICGs (index > 0) and its negative correlation (index < 0). **(B)** The forest plot of log 10 hazard ratios (HRs) with 95% CIs shows the relationship between 73 CD274-positively correlated ICGs and overall survival (OS). The vertical line represents a HR of 0. **(C)** The Gene Ontology analysis identifies the top 20 significant biological processes (BPs) enriched by the 8 prognostic ICGs positively correlated with CD274 (CTLA4, CD28, CD40LG, ADORA2A, B- and T-lymphocyte attenuator (BTLA), CD27, TNFRSF4, and ICOS). **(D)** Functional enrichment analysis identifies the top 20 significant KEGG signaling pathways enriched by the 8 prognostic ICGs positively correlated with CD274 (CTLA4, CD28, CD40LG, ADORA2A, BTLA, CD27, TNFRSF4, and ICOS).

The expression level of 88 ICGs in OSCC samples is shown in Figure 3. Supplementary Table S3 presents the expression level of 88 ICGs in oral tumor samples compared with healthy control oral samples. Among the 88 ICGs, 30 genes were found to be with the value of *p* < 0.05 and thus regarded as DEGs. Supplementary Table S4 presents the differential expression information of these 30 DEGs in oral tumor samples compared with healthy control oral samples. Figure 4A shows that 88 ICGs were mainly positively correlated. Interestingly, the aggregation effect was obviously observed, which indicated that the relationship between ICGs is mainly synergistic. It can be clearly observed that all of the other ICGs were positively correlated with CD274 except for four CD274-negatively correlated genes [i.e., DLX3 (Pearson correlation coefficient value *r* = −0.11), HHLA2 (*r* = −0.09), TNFRSF18 (*r* = −0.14), and VTCN1 (*r* = −0.02)]. This observation suggested that most ICGs play a coinhibitory role in tumor immunology and work synergistically with CD274, while the minority of ICGs play a costimulatory role in tumor immunology and work antagonistically with CD274. Because the correlation coefficient value |*r*| between CD274 and its negatively correlated ICGs were very small even <0.2 showing a poor correlation, thus only ICGs, which were positively correlated with CD274, were included in subsequent analysis.

### Identification of the Prognostic ICG That Is Significantly Positively Correlated With CD274

Among the 88 ICGs, 73 ICGs were selected based on the selection criteria: a positive correlation with CD274 and the value of *p* < 0.05. Based on the expression level of these 73 ICGs in the TCGA-OSCC data set and the prognosis of patients, Figure 4B shows the relationship between 73 ICGs and the prognosis using forest plots. The eight prognostic ICGs (CTLA4, ICOS, TNFRSF4, CD27, BTLA, ADORA2A, CD40LG, and CD28) with the value of *p* < 0.05 were marked with a five-pointed red star in Figure 4B. Supplementary Table S5 listed the hazard ratio- (HR-) related parameters (i.e., HR, HR with a lower/higher 95% confidence index) and the values of *p* of eight ICGs. Subsequent analysis was focused on these eight ICGs.

### Identification of Significantly Enriched Functional Terms of Eight ICGs

Figure 4C shows that the eight prognostic ICGs were significantly enriched in several TIICs related BPs, for example, T-cell-related BPs (e.g., T-cell proliferation, positive regulation of T-cell activation, T-cell costimulation, the regulation of T-cell activation, and T-cell activation), B-cell-related BPs (e.g., regulation of B-cell activation, and B-cell activation), lymphocyte-related BPs (e.g., the regulation of lymphocyte proliferation, lymphocyte costimulation, a positive regulation of lymphocyte activation, and the regulation of lymphocyte activation), and leukocyte-related BPs (e.g., a positive regulation of leukocyte cell–cell adhesion, leukocyte cell–cell adhesion, a positive regulation of leukocyte activation, the regulation of leukocyte cell–cell adhesion, and the regulation of leukocyte activation). In addition, the functional enrichment analysis results also revealed the significant KEGG pathways enriched by the eight prognostic ICGs, including the cytokine–cytokine receptor interaction, an immune network for IgA production, cell adhesion molecules, and a T-cell receptor signaling pathway (Figure 4D).

### Identification of TMB and Its Significantly Related ICGs From Eight ICGs

**Figure 5:**
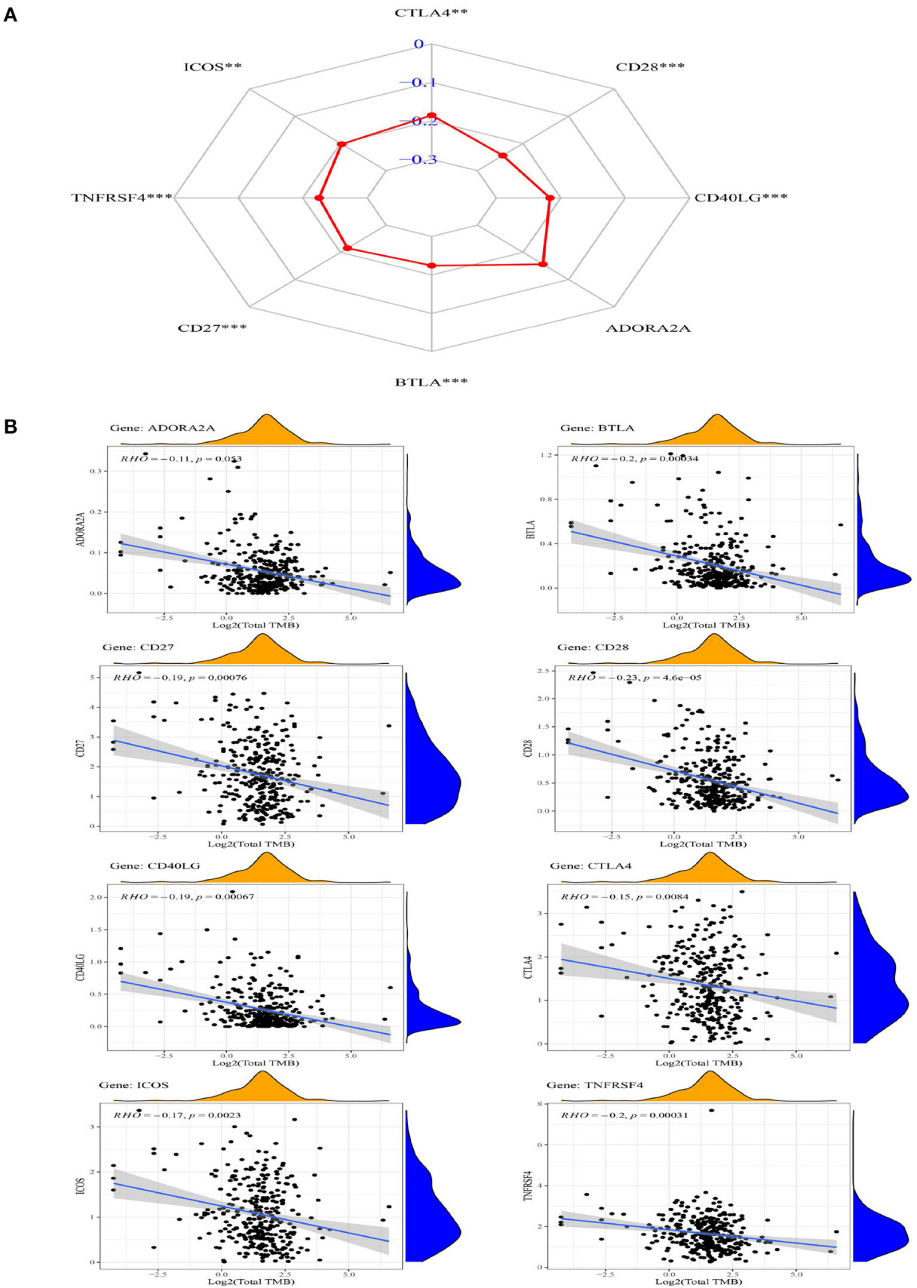
The radar chart **(A)** and scatter plot **(B)** showing the relationship between the eight ICGs positively correlated with CD274 (CTLA4, CD28, CD40LG, ADORA2A, BTLA, CD27, TNFRSF4, and ICOS) and TMB.

The correlation between TMB and eight ICGs identified in the abovementioned analysis was evaluated by using the Spearman correlation analysis. Supplementary Table S6 presents that the correlations between the expression of TMB and all of the eight ICGs were negative (< 0), and with a statistical significance (*p* < 0.05) except for ADORA2A. The results in Supplementary Table S6 are also displayed in the radar chart (Figure 5A) and also in the scatter plots (Figure 5B). These results showed that a high expression of these seven ICGs (CTLA4, ICOS, TNFRSF4, CD27, BTLA, CD40LG, and CD28) showing the worse/unfavorable prognosis corresponds to a low expression of TMB. TMB has been demonstrated to be a reliable biomarker for predicting the clinical efficacy of patients to PDL1 inhibitors (25). A low TMB predicts a poor response to PDL1 inhibitor therapy. The results in Supplementary Table S6, Figures 5A,B indicate that the patients with OSCC having a high expression of these seven ICGs are not suitable for the immune treatment of PDL1 inhibitor drugs and vice versa.

### Identification of ICGS Correlated With CD274 and Significantly Related to Clinical Features

**Figure 6:**
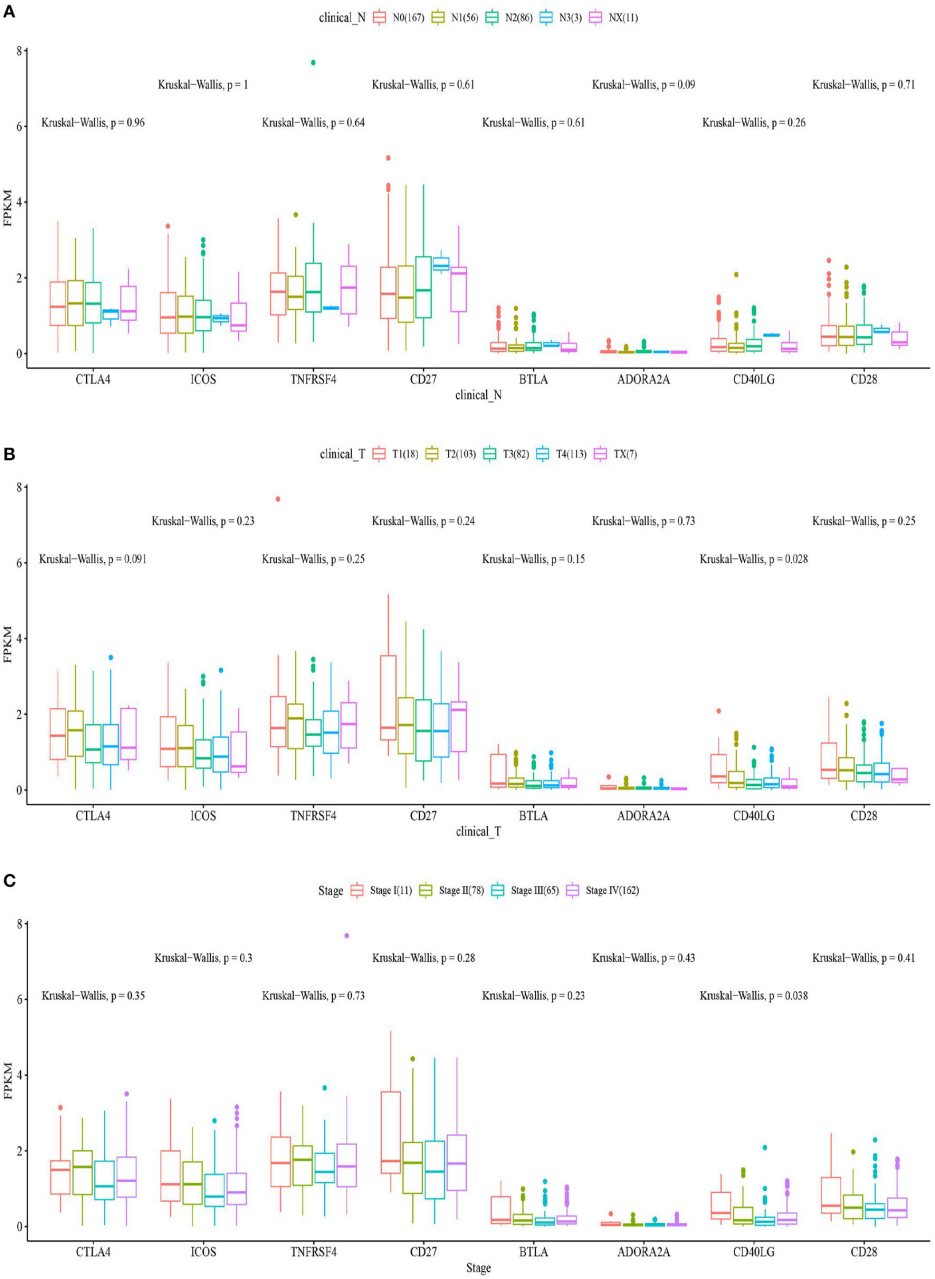
The box plots show the eight ICGs positively correlated with CD274 and clinical features including N **(A)**, T **(B)**, and stages **(C)**.

The box plots in Figure 6 show the relationship between the clinical features and eight ICGs positively correlated with CD274. Regarding the expression level, the eight ICGs correlated with CD274 were significantly divided into a high- and a low-expression group. The high-expression group consisted of CTLA4, ICOS, TNFRSF4, CD27, and the low-expression group consisted of BTLA, ADORA2A, CD40LG, and CD28. As shown in Figure 6A, in the significance test, no significance was found between any of the eight ICGs with N (nodes) (Kruskal–Wallis test, *p* > 0.05). As shown in Figure 6B, a significance (Kruskal–Wallis test, *p* = 0.028) was found between CD40LG and the T stage. As shown in Figure 6C, a significance (Kruskal–Wallis test, *p* = 0.038) was found between CD40LG and the clinical stage. Taken together, the majority of ICGs positively correlated with CD274 were not significantly correlated with clinical features.

### A Correlation Between CD274-Related ICGs and Adaptive Immune Resistance Pathway Genes

**Figure 7:**
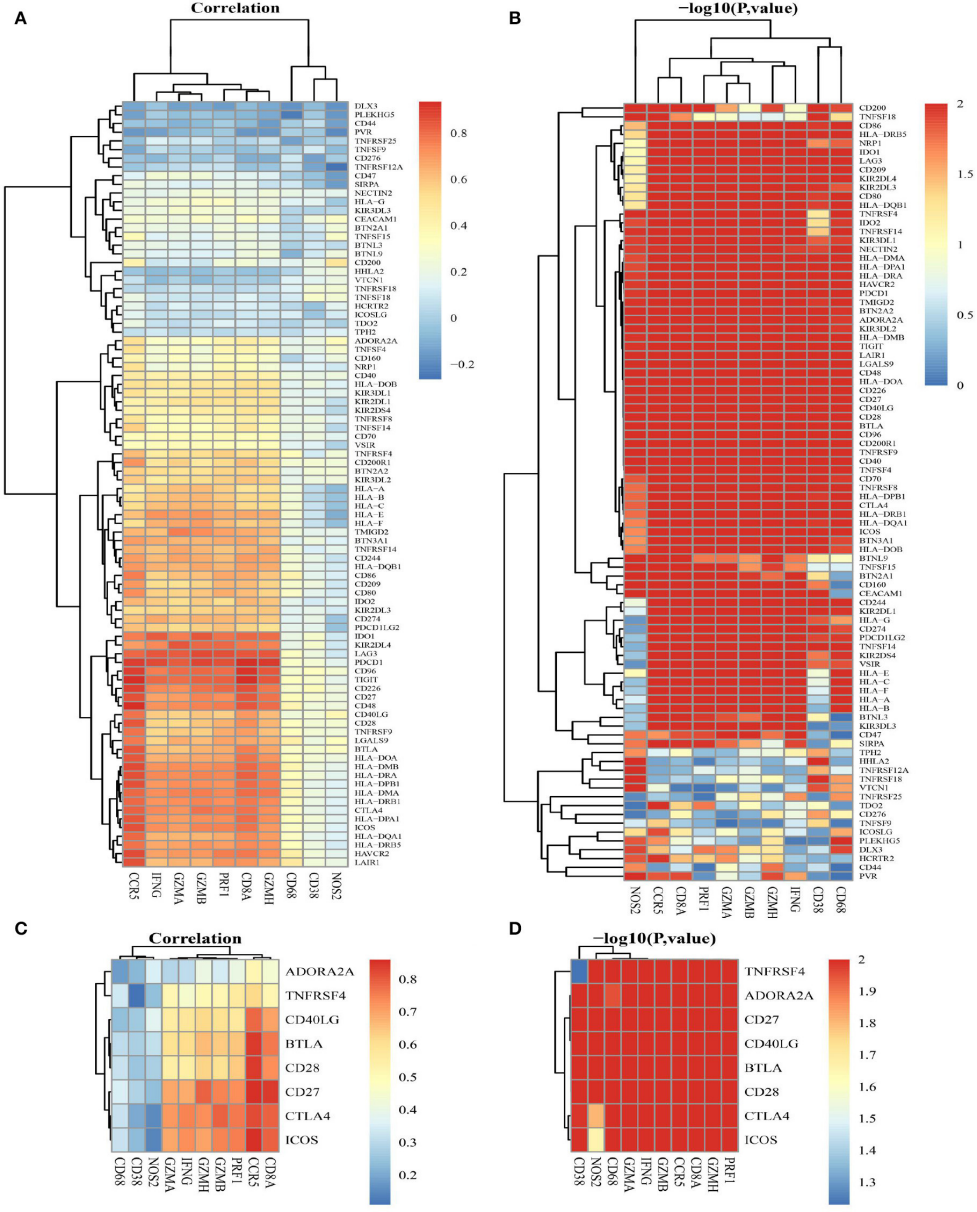
The correlation between adaptive immune resistance pathway genes and ICGs. **(A)** A heat map shows the correlation coefficients between 10 adaptive immune resistance pathway genes and 88 ICGs. **(B)** A heat map shows –log 10 *p* for the correlation between adaptive immune resistance pathway genes and 88 ICGs. **(C)** A heat map shows the correlation coefficients between adaptive immune resistance pathway genes and CD274-related ICGs (ADORA2A, TNFRSF4, CD40LG, BTLA, CD28, CD27, CTLA4, and ICOS). **(D)** A heat map shows –log 10 *p* for the correlation between adaptive immune resistance pathway genes and CD274-related ICGs (ADORA2A, TNFRSF4, CD40LG, BTLA, CD28, CD27, CTLA4, and ICOS).

Correlations between ICGs and 10 adaptive immune resistance pathway genes (CD68, NOS2, PRF1, GZMA, GZMB, GZMH, IFNG, CD8A, CD38, and CCR5) were analyzed. The heat map in Figures 7A,B show the correlation coefficients and –log 10 (*p*-value) between 10 adaptive immune resistance pathway genes and 88 ICGs, respectively. In Figures 7C,D, a heat map is used to particularly show the correlation coefficients and –log10 (*p*-value) between 10 adaptive immune resistance pathway genes and CD274-related ICGs. As shown in Figure 7, the majority of adaptive immune resistance pathway genes (e.g., PRF1, GZMA, GZMB, GZMH, IFNG, CD8A, and CCR5) were positively correlated with the expression of the CD274-related ICGs and the majority of ICGs, whereas the three adaptive immune resistance pathway genes (e.g., NOS2, CD38, and CD68) were negatively correlated to the expression of nine CD274-related ICGs and the majority of ICGs. As observed in Figures 7A,B, almost all of the correlations were significant by performing the significance test of correlation coefficients (log-rank *p* < 0.01).

### The Prognostic Values of the OSCC Subtypes Defined by ICGs Positively Correlated With CD274

**Figure 8:**
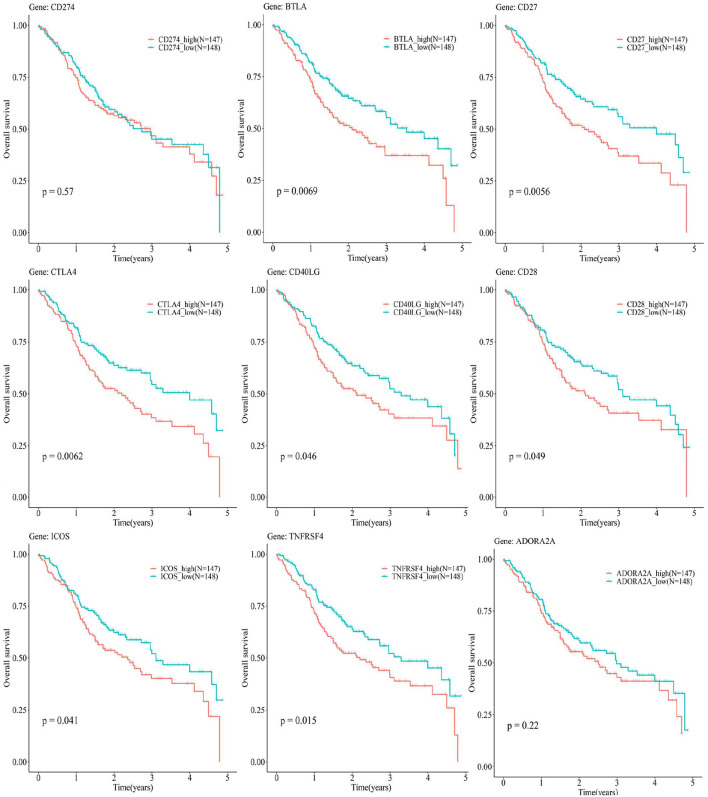
The Kaplan–Meier (KM) plots show the relationship between the 5-year OS and CD274-related ICGs [CD274 and its eight positively correlated ICGs (BTLA, CD27, CTLA4, CD40LG, CD28, ICOS, TNFRSF4, and ADORA2A)]. The red curves indicate the low-expression group, whereas the blue curves indicate the high-expression group.

Figure 8, Supplementary Table S7 show a relationship between the OS rate and CD274-related ICGs (CD274 and its positively correlated ICGs). Although there is no significant relationship between CD274 and the OS of OSCC [CD274 (*p* = 0.57 > 0.05)], the significant prognostic values of ICGs positively correlated with CD274 were observed, for example, BTLA (*p* = 0.0069 < 0.05), CD27 (*p* = 0.0056 < 0.05), CTLA4 (*p* = 0.0062 < 0.05), CD40LG (*p* = 0.046 < 0.05), CD28 (*p* = 0.049 < 0.05), ICOS (*p* = 0.041 < 0.05), and TNFRSF4 (*p* = 0.015 < 0.05).

### The Prognostic Values of the OSCC Subtypes Defined by CD274 and Its Positively Correlated ICGs

**Figure 9:**
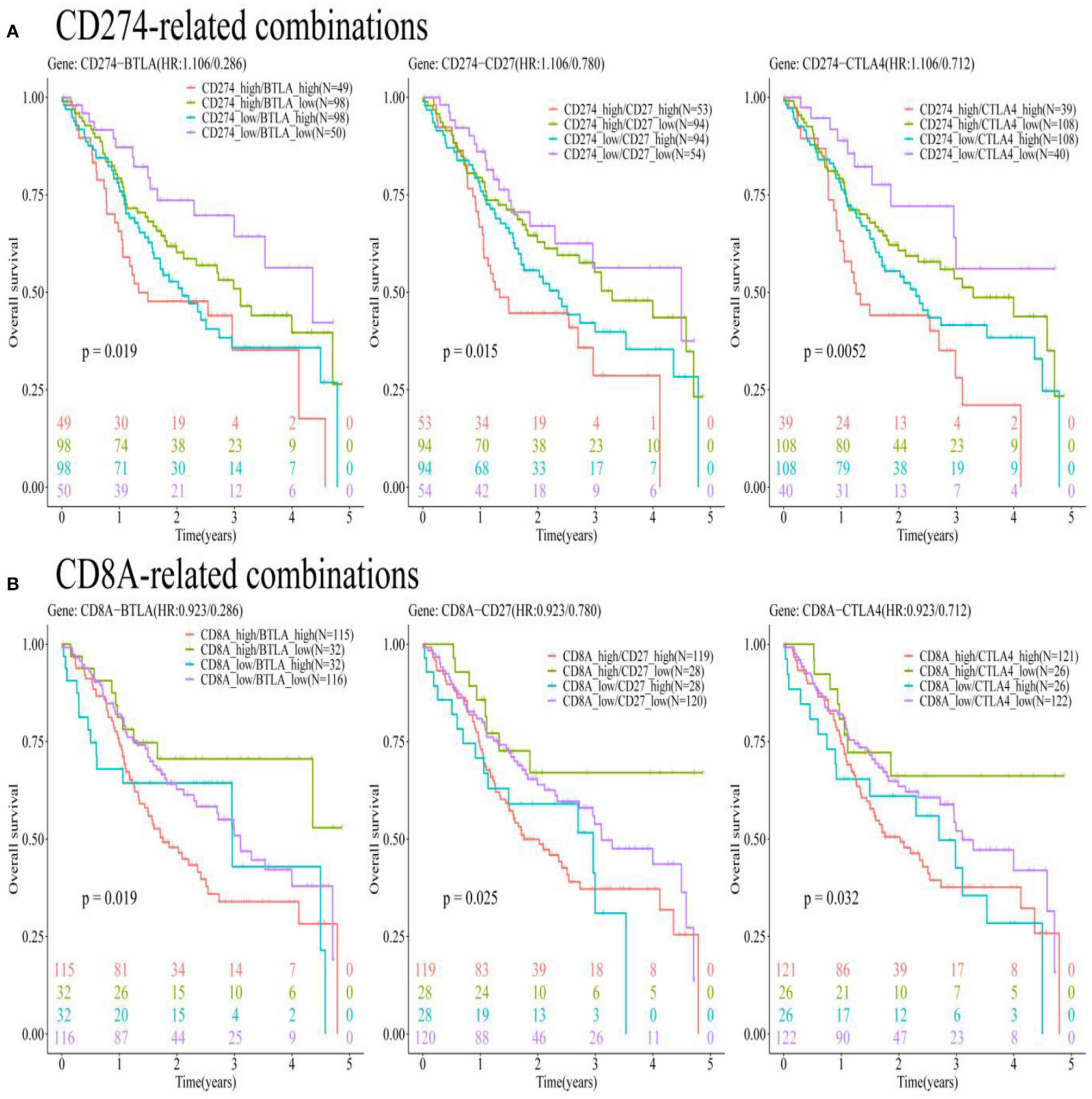
The KM plots show a significant relationship between the 5-year OS and gene pairs subtypes consisted of CD274-related genes, based on the TCGA-OSCC data analysis. **(A)** The KM plots show the prognostic values of subtypes consisted of CD274 and its positively correlated ICGs (BTLA, CD27, and CTLA4). **(B)** The KM plots show the prognostic values of CD8A and three ICGs positively correlated with CD274 (BTLA, CD27, and CTLA4).

Oral and squamous cell carcinoma samples were divided into four combinations based on the median value of gene expression levels for eight pairs of genes (CD274-CTLA4, CD274-ICOS, CD274-TNFRSF4, CD274-CD27, CD274-BTLA, CD274-ADORA2A, CD274-CD40LG, and CD274-CD28). Supplementary Table S8 presents that among these eight pairs of ICGs, only three pairs were found to be with significant prognostic values [CD274-BTLA (*p* = 0.019 < 0.05), CD274-CD27 (*p* = 0.015 < 0.05), and CD274-CTLA4 (*p* = 0.0052 < 0.05)]. Figure 9A shows the significance between the 5-year OS rate and the four high- and low-expression combinations for three pairs of genes (CD274-BTLA, CD274-CD27, and CD274-CTLA4).

### The Prognostic Values of the OSCC Subtypes Defined by CD8A and CD274-Related ICGs

Oral and squamous cell carcinoma samples were divided into four combinations based on the median value of gene expression levels for nine pairs of genes (CD8A-CD274, CD8A-CTLA4, CD8A-ICOS, CD8A-TNFRSF4, CD8A-CD27, CD8A-BTLA, CD8A-ADORA2A, CD8A-CD40LG, and CD8A-CD28). Supplementary Table S8 presents that among these nine pairs of ICGs, only four pairs were found to be with significant prognostic values [CD8A-BTLA (*p* = 0.019 <0.05), CD8A-CD27 (*p* = 0.025 < 0.05), CD274-CTLA4 (*p* = 0.032 < 0.05), and CD8A-TNFRSF4 (*p* = 0.031 < 0.05)]. To match the results in Figure 9A, only three pairs of genes (CD8A-BTLA, CD8A-CD27, and CD8A-CTLA4) were listed in Figure 9B to show the significance between the 5-year OS rate and the four high- and low-expression combinations corresponding to these three pairs of genes.

### Validation of the Prognostic Values of the Immune Subtypes

Four independent cohorts (i.e., GSE41613, GSE42743, GSE75538, and GSE85446) in the GEO database were used to verify the prediction accuracy of the prognostic values of the immune subtypes identified by the survival analysis based on the TCGA data. Supplementary Figures S1–S4 show the survival analysis results based on the four independent GEO data sets. These validation results were unfortunately not as anticipated and displayed insignificant prognostic values (*p* > 0.05) for the relationship between the OS and majority of immune subtype combinations identified by the TCGA-OSCC data set. This finding may be attributed to the small sample size of these GEO data sets as compared with the TCGA-OSCC data set [GSE41613: *n* = 42 samples, GSE42743 (*n* = 52), GSE75538 (*n* = 8), and GSE85446 (*n* = 32), and TCGA_OSCC data set (*n* = 295)]. As OSCC-related mRNA sequencing experiments are typically based on larger sample sizes, a sufficient documentation of the OS information should be encouraged in future research. Most of the existing GEO data sets lacked the survival information, and a few GEO data sets with the survival information had small sample sizes. Furthermore, other types of prognostic outcomes such as disease-free survival, metastasis-free survival, and progression-free survival were also lacking.

## Discussion

The present research aimed to characterize the prognostic subtypes of OSCC based on CD274 and its positively correlated ICGs. Using the mRNA expression data from TCGA and an independent cohort GEO data set, it was shown that eight ICGs (CTLA4, ICOS, TNFRSF4, CD27, BTLA, ADORA2A, CD40LG, and CD28) work synergistically and are positively correlated with CD274. An important finding was that although both CD8A and CD274 were not significantly related to OS, six subytypes with favorable survival were identified based on the three ICGs positively correlated with CD274 (BTLA, CD27, and CTLA4). The six subtypes showing a better survival were CD274_low/BTLA_low, CD274_low/CD27_low, CD274_low/CTLA4_low, CD8A_high/BTLA_low, CD8A_high/CD27_low, and CD8A_high/CTLA4_low.

Based on the synergistic correlation and prognosis analysis, the three combination strategies of ICG inhibitors drugs may be suggested, including, an inhibitor of PDL1+ inhibitor of BTLA, inhibitor of PDL1+ inhibitor of CD27, and inhibitor of PDL1+ inhibitor of CTLA. Much previous evidence supports the findings of the current study. A high expression of BTLA in different types of cancers (e.g., colorectal cancer, melanoma cancer, and lung cancer) was found to inhibit the expression and function of T-cells (51–53). Patients with lung cancer negative for both BTLA and PDL1 showed a better relapse-free survival (RFS) compared with patients, positive for either BTLA or PDL1 (51). Although BTLA and PDL1 employ distinct phosphatases to suppress T-cell signaling, both of them dampen the TCR and CD28 signaling pathways equally, and thus the inhibitors of both BTLA and PDL1 might be regarded as a combination of immunotherapeutic agents for cancer treatment (54, 55). However, the data regarding BTLA in oral cancer were still lacking. The TNFR superfamily member CD27 showed a synergistic correlation with PDL1, and a low expression of CD27 indicated a significantly better survival outcome as compared with its high expression, and a low expression of both PDL1 and CD27 indicated the best survival. Contradictory results are reported in previous studies, showing CD27 as a costimulatory ICG, which plays critical roles in the activation, proliferation, and survival of T-cells, and that the blockade of PDL1 and agonist of CD27 activates CD8+ T-cell-driven antitumor immunity (56–58). Tumor heterogeneity and different cancer types may underlie these contradictory findings. The present research showed that a low expression of CTLA4 indicated a better prognosis. Moreover, a low expression of both PDL1 and CTLA4 indicated an improved prognosis. This is in line with available research. The inhibitor of CTLA4-ipilimumab has been approved by FDA for treating melanoma (59). The mechanistic aspects of PDL1 and CTLA4 vary in immuno-oncology and PDL1 plays a suppressing role at the later stage of immune response, whereas CTLA4 plays an inhibiting role at the early stage of immune response (60). However, anti-PDL1 and anti-CTLA4 treatments have additive and synergistic effects on cancer treatment, based on the observation of the superior efficacy obtained by using the combination of CTLA4 blockade and PDL1 blockade in patients with melanoma compared with monotherapy (61).

In addition, the subtyping findings based on CD8A suggested that the coevaluation of the CD8A expression level and three ICGs positively correlated with CD274 (BTLA, CD27, and CTLA4) may enable a better evaluation of the immunological state of OSCC. The current study showed that patients with OSCC having the best survival had an increased CD8A infiltration and a low expression of one of the three ICGs positively correlated with CD274 (BTLA, CD27, and CTLA4). These results are reasonable considering CD8A is a surface biomarker of effector T-cells, and CD8+ T-cells in the TME indicate a good prognosis in many cancer types (62). The low expression of three ICGs positively correlated with CD274 (BTLA, CD27, and CTLA4) indicated a better prognosis compared to their high expression, indicating their coinhibitory roles in the tumor immunology of oral cancer. A previous study using the bioinformatic analysis also provided similar results, showing that patients with pancreatic adenocarcinoma (PDAC) with the best survival had increased CD8A infiltration without the expression of CD274 (63). Although the current study did not find a statistical significance of the relationship between CD8A/CD274 immunotypes and prognosis, significant prognostic values of three ICGs positively correlated with CD8A/CD274 (BTLA, CD27, and CTLA4) were found. A high expression of BTLA indicated a poor prognosis of OSCC, which is in accordance with the results of a previous study on ovarian cancer (64). A previous examination regarding melanoma showed that BTLA+ CD8+ tumor-infiltrating lymphocytes (TILs) showed a superior response and better survival compared to BTLA- CD8+ TILs (65) as BTLA plays a costimulatory role on activating CD8+ T-cells in melanoma. Such results were contradictory with the current research, which showed that the BTLA- CD8+ subset had the best survival in oral cancer, and might be explained by varying the roles of BTLA in different cancers. Previous studies have shown a costimulatory role of CD27 in inducing a potent proliferation of CD8+ T-cells, with a significant production of Th1 cytokines (IFNα, TNFα, and IL-2) and Th2 cytokines (mainly IL-13) by T-cells (7). This is contradictory with the results in the current computational prediction regarding oral cancer, which may be attributed to CD27 acting as either a costimulatory or coinhibitory receptor in different cancers and different circumstances (66). Considering the combination of CTLA4-CD8A, the present research showed that patients with OSCC with high CD8A T-cell infiltration and a low expression of CTLA4 had the best prognosis. Previous research showed that the administration of CTLA4 blockade could increase the expansion and enhance the effector function of memory CD8+ T-cells, thereby contributing to the great accumulation of functional memory CD8+ T-cells (67). CTLA4+ tumor-infiltrating cells have been found to be an independent prognostic factor in OSCC, showing that its high infiltration indicated worse recurrence-free survival and metastasis-free survival (68).

It is important to clearly state the strengths and limitations of the current study. The main strength of the present study is that a series of comprehensive bioinformatics analyses was performed, including the analysis of TIICs, correlation analysis, TMB analysis, clinical feature relationship analysis, and survival analysis for identifying prognostic immune subtypes. The first limitation is the lack of experimental validation of the estimated synergistic effects of the administration of a PDL1 inhibitor and its positively correlated ICGs in OSCC. The second limitation is the lack of experimental validation of the prognostic values of the PDL1-based immune subtypes in OSCC. Both these aspects suggest the experimental design direction for future research. Another important limitation is the small sample size of the oral cancer-related data sets with the survival information for validation, which could be a reason for the insignificant results concerning the value of the immune subtypes. Although the findings of the analysis based on the TCGA data were statistically significant, the magnitude of their potential clinical effects must be recognized and could only be evaluated in clinical settings.

It is noteworthy to highlight the potential implications and the clinical transfer values of the current study. Firstly, the combination of PDL-1-based ICG inhibitors might play additive and synergistic roles in the immune therapy of OSCC as compared to using either one of them alone. Thereby, the combinations identified in the current study might indicate novel therapeutic strategies for oral cancer treatment. Secondly, the immune subtypes identified in the current study could be used for predicting the OS outcomes of patients with oral cancer, and chairside testing based on these subsets could be developed as a useful prognostic prediction tool. Most importantly, the prognostic immune subtypes identified in the current study can have clinical implications for personalized immunotherapy. Patients belonging to specific subtypes should be administered with suitable ICG inhibitor agents with maximal efficacy. For example, considering the PDL1+CTLA4+ subset, this subgroup of patients with OSCC can receive a combination of a PDL1 inhibitor and CTLA4 inhibitor. However, the PDL1 and CTLA4 inhibitor agents will be not useful for the PDL1-CTLA4- subset. Therefore, immune subtypes as identified in this study can guide a refined patient selection, and enable personalized immune therapy strategies to significantly improve the OS in OSCC.

## Conclusion

The current study identified several ICGs positively correlated with CD274 (BLTA, CD27, and CTLA4) comprising immune subtypes indicating favorable OS outcomes, including CD274_low/BTLA_low, CD274_low/CD27_low, CD274_low/CTLA4_low, CD8A_high/BTLA_low, CD8A_high/CD27_low, and CD8A_high/CTLA4_low. These findings suggest that the three combinations of ICG inhibitors might play synergistic and additive effects in treating and improving the prognosis of OSCC, i.e., the combination of a CD274 inhibitor and BTLA inhibitor, the combination of a CD274 inhibitor and CD27 inhibitor, and the combination of a CD274 inhibitor and CTLA4 inhibitor. The combination of immune subtypes and suggested drug agents might provide precise immune strategies for application in personalized oral cancer treatment.

## Data Availability Statement

The original contributions presented in the study are included in the article/Supplementary Material, further inquiries can be directed to the corresponding author/s.

## Author Contributions

YY and HT contributed to the conceptualization, methodology, formal analysis, and writing—original draft. DF, PM, AA, and YD contributed to the data curation, formal analysis, methodology, resources, software, and visualization. BL, VS, RZ, and DZ contributed to the formal analysis, methodology, writing—review and editing. YY, SL, and GS contributed to the project administration, supervision, writing—review and editing. All authors contributed to the article and approved the submitted version.

## Funding

We appreciate the funding provided by the Science Research Cultivation Program of Stomatological Hospital, Southern Medical University (No. PY2020004) to support the postdoctoral research of the senior author Dr. SL (simin.li.dentist@gmail.com).

## Conflict of Interest

The authors declare that the research was conducted in the absence of any commercial or financial relationships that could be construed as a potential conflict of interest.

## Publisher's Note

All claims expressed in this article are solely those of the authors and do not necessarily represent those of their affiliated organizations, or those of the publisher, the editors and the reviewers. Any product that may be evaluated in this article, or claim that may be made by its manufacturer, is not guaranteed or endorsed by the publisher.
