# Supplementary material for: Immune Checkpoint Gene Expression Profiling Identifies Programmed Cell Death Ligand-1 Centered Immunologic Subtypes of Oral and Squamous Cell Carcinoma With Favorable Survival
Source: Front Med (Lausanne). 2022 Jan 20;8:759605. doi: 10.3389/fmed.2021.759605 (PMC8810827; doi:10.3389/fmed.2021.759605)
Supplement: Supplementary file 1 [file Data_Sheet_1.docx]

**Supplementary Materials**

**Table S1.** The list of ICGs which were investigated in the present study.

| **ICG genes** | **Other names** | **Full Name** | **webpage** |
| --- | --- | --- | --- |
| **PD1** | PDCD1; PD-1; CD279; SLEB2; hPD-1; hPD-l; Hsle1 | programmed cell death 1 | https://www.ncbi.nlm.nih.gov/gene/5133 |
| **PDL1** | CD274; B7-H; B7H1; PD-L1; hPD-L1; PDCD1L1; PDCD1LG1 | CD274 molecule; programmed cell death 1 ligand 1 | https://www.ncbi.nlm.nih.gov/gene/29126 |
| **PDL2** | PDCD1LG2 | programmed cell death 1 ligand 2 | https://www.ncbi.nlm.nih.gov/gene/80380 |
| **ICOS** | AILIM; CD278; CVID1 | inducible T cell costimulator | https://www.ncbi.nlm.nih.gov/gene/29851 |
| **ICOSLG** | B7h; B7H2; GL50; B7-H2; B7RP1; CD275; ICOSL; LICOS; B7RP-1; ICOS-L | inducible T cell costimulator ligand | https://www.ncbi.nlm.nih.gov/gene/23308 |
| **BTN2A2** | BTF2; BT2.2; BTN2.2 | butyrophilin subfamily 2 member A2 | https://www.ncbi.nlm.nih.gov/gene/10385 |
| **BTN2A1** | BTF1; BT2.1; BTN2.1; DJ3E1.1; BK14H9.1 | butyrophilin subfamily 2 member A1 | https://www.ncbi.nlm.nih.gov/gene/11120 |
| **BTN3A1** | BTF5; BT3.1; CD277; BTN3.1 | butyrophilin subfamily 3 member A1 | https://www.ncbi.nlm.nih.gov/gene/11119 |
| **BTNL3** | BTNLR; BTN9.1 | butyrophilin like 3 | https://www.ncbi.nlm.nih.gov/gene/10917 |
| **BTNL9** | BTN3; BTN8; VDLS1900 | butyrophilin like 9 | https://www.ncbi.nlm.nih.gov/gene/153579 |
| **B7-H3** | CD276 | CD276 molecule | https://www.ncbi.nlm.nih.gov/gene/80381 |
| **B7-H4** | VTCN1; B7X; B7H4; B7S1; B7-H4; B7h.5; VCTN1; PRO1291 | V-set domain containing T cell activation inhibitor 1 | https://www.ncbi.nlm.nih.gov/gene/79679 |
| **IDO1** | IDO; INDO; IDO-1 | indoleamine 2,3-dioxygenase 1 | https://www.ncbi.nlm.nih.gov/gene/3620 |
| **IDO2** | INDOL1 | indoleamine 2,3-dioxygenase 2 | https://www.ncbi.nlm.nih.gov/gene/169355 |
| **CD200** | MRC; MOX1; MOX2; OX-2 | CD200 molecule | https://www.ncbi.nlm.nih.gov/gene/4345 |
| **CD200R** | CD200R1; OX2R; MOX2R; HCRTR2 | CD200 receptor 1 | https://www.ncbi.nlm.nih.gov/gene/131450 |
| **CD223** | LAG3 | lymphocyte activating 3 | https://www.ncbi.nlm.nih.gov/gene/3902 |
| **CD244** | 2B4; NAIL; Nmrk; NKR2B4; SLAMF4 | CD244 molecule | https://www.ncbi.nlm.nih.gov/gene/51744 |
| **CD80** | B7; BB1; B7-1; B7.1; LAB7; CD28LG; CD28LG1 | CD80 molecule | https://www.ncbi.nlm.nih.gov/gene/941 |
| **CD86** | B70; B7-2; B7.2; LAB72; CD28LG2 | CD86 molecule | https://www.ncbi.nlm.nih.gov/gene/942 |
| **CD28** | Tp44 | CD28 molecule | https://www.ncbi.nlm.nih.gov/gene/940 |
| **CD160** | NK1; BY55; NK28 | CD160 molecule | https://www.ncbi.nlm.nih.gov/gene/11126 |
| **CD47** | IAP; OA3; MER6 | CD47 molecule | https://www.ncbi.nlm.nih.gov/gene/961 |
| **CD244** | 2B4; CD244; NAIL; Nmrk; NKR2B4; SLAMF4 | CD244 molecule | https://www.ncbi.nlm.nih.gov/gene/51744 |
| **CD48** | BCM1; BLAST; hCD48; mCD48; BLAST1; SLAMF2; MEM-102 | CD48 molecule | https://www.ncbi.nlm.nih.gov/gene/962 |
| **CD209** | DC-SIGN; CDSIGN; CLEC4L; DC-SIGN1 | CD209 molecule | https://www.ncbi.nlm.nih.gov/gene/30835 |
| **CD226** | DNAM1; CD226; PTA1; DNAM-1; TLiSA1 | CD226 molecule | https://www.ncbi.nlm.nih.gov/gene/10666 |
| **CD155** | PVR; PVS; HVED; NECL5; TAGE4; Necl-5 | PVR cell adhesion molecule | https://www.ncbi.nlm.nih.gov/gene/5817 |
| **CD305** | LAIR1; CD305; LAIR-1 | leukocyte associated immunoglobulin like receptor 1 | https://www.ncbi.nlm.nih.gov/gene/3903 |
| **CD44** | IN; LHR; MC56; MDU2; MDU3; MIC4; Pgp1; CDW44; CSPG8; HCELL; HUTCH-I; ECMR-III | CD44 molecule (Indian blood group) | https://www.ncbi.nlm.nih.gov/gene/960 |
| **CD96** | TACTILE | CD96 molecule | https://www.ncbi.nlm.nih.gov/gene/10225 |
| **CD112** | NECTIN2; HVEB; PRR2; CD112; PVRL2; PVRR2 | nectin cell adhesion molecule 2 | https://www.ncbi.nlm.nih.gov/gene/5819 |
| **CD304** | NRP1; NP1; NRP; BDCA4; CD304; VEGF165R | neuropilin 1 | https://www.ncbi.nlm.nih.gov/gene/8829 |
| **CD272** | BTLA; BTLA1; CD272 | B and T lymphocyte associated | https://www.ncbi.nlm.nih.gov/gene/151888 |
| **CTLA4** | CTLA-4 | cytotoxic T-lymphocyte associated protein 4 | https://www.ncbi.nlm.nih.gov/gene/1493 |
| **TIGIT** | VSIG9; VSTM3; WUCAM | T cell immunoreceptor with Ig and ITIM domains | https://www.ncbi.nlm.nih.gov/gene/201633 |
| **ADORA2A** | A2aR; RDC8; ADORA2 | adenosine A2a receptor | https://www.ncbi.nlm.nih.gov/gene/135 |
| **TIM-3** | HAVCR2 | hepatitis A virus cellular receptor 2 | https://www.ncbi.nlm.nih.gov/gene/84868 |
| **VISTA** | VSIR; B7H5; GI24; B7-H5; Dies1; PD-1H; SISP1; VISTA; PP2135; C10orf54; DD1alpha | V-set immunoregulatory receptor | https://www.ncbi.nlm.nih.gov/gene/64115 |
| **LGALS9** | HUAT; LGALS9A; Gal-9 | galectin 9 | https://www.ncbi.nlm.nih.gov/gene/3965 |
| **CEACAM1** | BGP; BGP1; BGPI | CEA cell adhesion molecule 1 | https://www.ncbi.nlm.nih.gov/gene/634 |
| **SIRPA** | BIT; MFR; P84; SIRP; MYD-1; SHPS1; CD172A; PTPNS1 | signal regulatory protein alpha | https://www.ncbi.nlm.nih.gov/gene/140885 |
| **TMIGD2** | CD28H; IGPR1; IGPR-1 | transmembrane and immunoglobulin domain containing 2 | https://www.ncbi.nlm.nih.gov/gene/126259 |
| **HHLA2** | B7y; B7H7; B7-H5; B7-H7 | HERV-H LTR-associating 2 | https://www.ncbi.nlm.nih.gov/gene/11148 |
| **TDO** | DLX3; AI4 | distal-less homeobox 3 | https://www.ncbi.nlm.nih.gov/gene/1747 |
| **TDO2** | TO; TDO; TPH2; TRPO; HYPTRP | tryptophan 2,3-dioxygenase | https://www.ncbi.nlm.nih.gov/gene/6999 |
| **TNFRSF25** | DR3; TR3; DDR3; LARD; APO-3; TRAMP; WSL-1; GEF720; WSL-LR; PLEKHG5; TNFRSF12 | TNF receptor superfamily member 25 | https://www.ncbi.nlm.nih.gov/gene/8718 |
| **TNFRSF8** | CD30; Ki-1; D1S166E | TNF receptor superfamily member 8 | https://www.ncbi.nlm.nih.gov/gene/943 |
| **TNFRSF12A** | FN14; CD266; TWEAKR | TNF receptor superfamily member 12A | https://www.ncbi.nlm.nih.gov/gene/51330 |
| **TNFSF15** | TL1; TL1A; VEGI; TNLG1B; VEGI192A | TNF superfamily member 15 | https://www.ncbi.nlm.nih.gov/gene/9966 |
| **TNFSF4** | GP34; CD252; OX4OL; TXGP1; CD134L; OX-40L; OX40L; TNLG2B | TNF superfamily member 4 | https://www.ncbi.nlm.nih.gov/gene/7292 |
| **TNFRSF4** | OX40; ACT35; CD134; IMD16; TXGP1L | TNF receptor superfamily member 4 | https://www.ncbi.nlm.nih.gov/gene/7293 |
| **TNFSF18** | GITRL; TL6; AITRL; TNLG2A; hGITRL | TNF superfamily member 18 | https://www.ncbi.nlm.nih.gov/gene/8995 |
| **TNFRSF18** | AITR; GITR; CD357; GITR-D; ENERGEN | TNF receptor superfamily member 18 | https://www.ncbi.nlm.nih.gov/gene/8784 |
| **TNFSF14** | LIGHT; LTg; CD258; HVEML | TNF superfamily member 14 | https://www.ncbi.nlm.nih.gov/gene/8740 |
| **TNFRSF14** | HVEM; TR2; ATAR; HVEA; HVEM; CD270; LIGHTR | TNF receptor superfamily member 14 | https://www.ncbi.nlm.nih.gov/gene/8764 |
| **TNFSF9** | CD137L; TNLG5A; 4-1BB-L | TNF superfamily member 9 | https://www.ncbi.nlm.nih.gov/gene/8744 |
| **TNFRSF9** | CD137; ILA; 4-1BB | TNF receptor superfamily member 9 | https://www.ncbi.nlm.nih.gov/gene/3604 |
| **TNFSF7** | CD70; CD27L; LPFS3; CD27-L; CD27LG; TNLG8A | TNF Superfamily Member 7 | https://www.ncbi.nlm.nih.gov/gene/970 |
| **TNFRSF7** | CD27; T14; S152; Tp55; S152. LPFS2 | TNF Receptor Superfamily Member 7 | https://www.ncbi.nlm.nih.gov/gene/939 |
| **TNFSF5** | CD40LG; IGM; IMD3; TRAP; gp39; CD154; CD40L; HIGM1; T-BAM; hCD40L | TNF Superfamily Member 5 | https://www.ncbi.nlm.nih.gov/gene/959 |
| **TNFRSF5** | CD40; p50; Bp50; CDW40 | TNF Receptor Superfamily Member 5 | https://www.ncbi.nlm.nih.gov/gene/958 |
| **HLA-G** | MHC-G | major histocompatibility complex, class I, G | https://www.ncbi.nlm.nih.gov/gene/3135 |
| **HLA-A** | HLAA | major histocompatibility complex, class I, A | https://www.ncbi.nlm.nih.gov/gene/3105 |
| **HLA-B** | AS; HLAB; B-4901 | major histocompatibility complex, class I, B | https://www.ncbi.nlm.nih.gov/gene/3106 |
| **HLA-C** | MHC; HLAC; HLC-C; D6S204; PSORS1; HLA-JY3 | major histocompatibility complex, class I, C | https://www.ncbi.nlm.nih.gov/gene/3107 |
| **HLA-E** | QA1; HLA-6.2 | major histocompatibility complex, class I, E | https://www.ncbi.nlm.nih.gov/gene/3133 |
| **HLA-F** | HLAF; CDA12; HLA-5.4; HLA-CDA12 | major histocompatibility complex, class I, F | https://www.ncbi.nlm.nih.gov/gene/3134 |
| **HLA-DMA** | DMA; HLADM; RING6; D6S222E | major histocompatibility complex, class II, DM alpha | https://www.ncbi.nlm.nih.gov/gene/3108 |
| **HLA-DMB** | RING7; D6S221E | major histocompatibility complex, class II, DM beta | https://www.ncbi.nlm.nih.gov/gene/3109 |
| **HLA-DOA** | HLADZ; HLA-DNA; HLA-DZA | major histocompatibility complex, class II, DO alpha | https://www.ncbi.nlm.nih.gov/gene/3111 |
| **HLA-DOB** | DOB; HLA_DOB | major histocompatibility complex, class II, DO beta | https://www.ncbi.nlm.nih.gov/gene/3112 |
| **HLA-DPA1** | DPA1; PLT1; HLADP; HLASB; DP(W3); DP(W4); HLA-DP1A | major histocompatibility complex, class II, DP alpha 1 | https://www.ncbi.nlm.nih.gov/gene/3113 |
| **HLA-DPB1** | DPB1; HLA-DP; HLA-DPB; HLA-DP1B | major histocompatibility complex, class II, DP beta 1 | https://www.ncbi.nlm.nih.gov/gene/3115 |
| **HLA-DQA1** | DQA1; DQ-A1; CELIAC1; HLA-DQA | major histocompatibility complex, class II, DQ alpha 1 | https://www.ncbi.nlm.nih.gov/gene/3117 |
| **HLA-DQB1** | IDDM1; CELIAC1; HLA-DQB | major histocompatibility complex, class II, DQ beta 1 | https://www.ncbi.nlm.nih.gov/gene/3119 |
| **HLA-DRA** | HLA-DRA1 | major histocompatibility complex, class II, DR alpha | https://www.ncbi.nlm.nih.gov/gene/3122 |
| **HLA-DRB1** | SS1; DRB1; HLA-DRB; HLA-DR1B | major histocompatibility complex, class II, DR beta 1 | https://www.ncbi.nlm.nih.gov/gene/3123 |
| **HLA-DRB3** | DRB3; HLA-DPB1; HLA-DR1B; HLA-DR3B; HLA-DRB3* | major histocompatibility complex, class II, DR beta 3 | https://www.ncbi.nlm.nih.gov/gene/3125 |
| **HLA-DRB4** | DR4; DRB4; HLA-DR4B; HLA-DRB4* | major histocompatibility complex, class II, DR beta 4 | https://www.ncbi.nlm.nih.gov/gene/3126 |
| **HLA-DRB5** | HLA-DRB5* | major histocompatibility complex, class II, DR beta 5 | https://www.ncbi.nlm.nih.gov/gene/3127 |
| **KIR3DL1** | KIR; NKB1; NKAT3; NKB1B; NKAT-3; CD158E1; KIR2DL5B; KIR3DL1/S1 | killer cell immunoglobulin like receptor, three Ig domains and long cytoplasmic tail 1 | https://www.ncbi.nlm.nih.gov/gene/3811 |
| **KIR2DL1** | NKAT; NKAT1; p58.1; CD158A; KIR221; NKAT-1; KIR-K64; KIR2DL3 | killer cell immunoglobulin like receptor, two Ig domains and long cytoplasmic tail 1 | https://www.ncbi.nlm.nih.gov/gene/3802 |
| **KIR2DL2** | NKAT6; p58.2; CD158b; NKAT-6; CD158B1 | killer cell immunoglobulin like receptor, two Ig domains and long cytoplasmic tail 2 | https://www.ncbi.nlm.nih.gov/gene/3803 |
| **KIR2DL3** | p58; NKAT; GL183; NKAT2; CD158b; KIR2DL; NKAT2A; NKAT2B; CD158B2; KIR-K7b; KIR-K7c; KIR2DS5; KIRCL23; KIR-023GB | killer cell immunoglobulin like receptor, two Ig domains and long cytoplasmic tail 3 | https://www.ncbi.nlm.nih.gov/gene/3804 |
| **KIR2DL4** | G9P; CD158D; KIR103; KIR-2DL4; KIR103AS; KIR-103AS | killer cell immunoglobulin like receptor, two Ig domains and long cytoplasmic tail 4 | https://www.ncbi.nlm.nih.gov/gene/3805 |
| **KIR2DS2** | NKAT5; cl-49; CD158J; CD158b; NKAT-5; 183ActI; KIR2DL1; KIR-2DS2 | killer cell immunoglobulin like receptor, two Ig domains and short cytoplasmic tail 2 | https://www.ncbi.nlm.nih.gov/gene/100132285 |
| **KIR3DL2** | 3DL2; p140; NKAT4; CD158K; NKAT-4; NKAT4B; KIR-3DL2 | killer cell immunoglobulin like receptor, three Ig domains and long cytoplasmic tail 2 | https://www.ncbi.nlm.nih.gov/gene/3812 |
| **KIR2DL5A** | CD158F; KIR2DL5; KIR2DL5.1; KIR2DL5.3 | killer cell immunoglobulin like receptor, two Ig domains and long cytoplasmic tail 5A | https://www.ncbi.nlm.nih.gov/gene/57292 |
| **KIR2DS1** | p50.1; CD158H; CD158a | killer cell immunoglobulin like receptor, two Ig domains and short cytoplasmic tail 1 | https://www.ncbi.nlm.nih.gov/gene/3806 |
| **KIR2DS4** | KKA3; KIR1D; NKAT8; CD158I; KIR412; NKAT-8; KIR-2DS4 | killer cell immunoglobulin like receptor, two Ig domains and short cytoplasmic tail 4 | https://www.ncbi.nlm.nih.gov/gene/3809 |
| **KIR2DS5** | NKAT9; CD158G | killer cell immunoglobulin like receptor, two Ig domains and short cytoplasmic tail 5 | https://www.ncbi.nlm.nih.gov/gene/3810 |
| **KIR3DL3** | KIR44; KIRC1; CD158Z; KIR3DL7 | killer cell immunoglobulin like receptor, three Ig domains and long cytoplasmic tail 3 | https://www.ncbi.nlm.nih.gov/gene/115653 |
| **KIR3DS1** | KIR-G1; NKAT10; CD158E2; NKAT-10; KIR-123FM | killer cell immunoglobulin like receptor, three Ig domains and short cytoplasmic tail 1 | https://www.ncbi.nlm.nih.gov/gene/3813 |
| **KIR2DS3** | NKAT7 | killer cell immunoglobulin like receptor, two Ig domains and short cytoplasmic tail 3 | https://www.ncbi.nlm.nih.gov/gene/3808 |

**Table S2.** The sample size belonging to the different anatomic sites of 326 OSCC samples in the TCGA-HNSCC data.

| Site Of Biopsy Diagnoses | Sample Size |
| --- | --- |
| Anterior floor of mouth | 2 |
| Base of tongue | 22 |
| Border of tongue | 1 |
| Cheek mucosa | 19 |
| Floor of mouth | 55 |
| Gum | 8 |
| Hard palate | 4 |
| Lower gum | 2 |
| Mandible | 1 |
| Mouth | 21 |
| Overlapping lesion of lip | 82 |
| Palate | 1 |
| Retromolar area | 1 |
| Tongue | 138 |
| Upper gum | 1 |

**Table S3.** The differential expression information of 88 ICGs in OSCC samples compared with healthy control samples.

| **genes** | **logFC** | **logCPM** | **F** | **PValue** |
| --- | --- | --- | --- | --- |
| **VTCN1** | 1.204670031 | 13.74045104 | 24.38987235 | 1.20E-06 |
| **CEACAM1** | 1.187418948 | 14.28389901 | 102.4626474 | 8.83E-15 |
| **TNFSF15** | 1.046883771 | 13.60919238 | 55.11694022 | 9.87E-05 |
| **CD200R1** | 0.952862188 | 13.5390411 | 54.37996505 | 0.001273126 |
| **CD40LG** | 0.940153382 | 13.4754551 | 37.39746348 | 0.003326497 |
| **BTNL9** | 0.414793985 | 13.59236316 | 2.895946043 | 0.173330144 |
| **HHLA2** | 0.384881249 | 13.31965513 | 6.348040143 | 0.307553104 |
| **CD48** | 0.308382609 | 14.12144711 | 8.238085406 | 0.121673774 |
| **HLA-DOA** | 0.299427092 | 14.30911215 | 7.667177398 | 0.090947784 |
| **VSIR** | 0.283387027 | 14.83205828 | 26.6519737 | 0.034237965 |
| **HLA-DPB1** | 0.271958117 | 15.17744142 | 66.66324327 | 0.017027459 |
| **CD200** | 0.270017509 | 14.05558385 | 3.624083247 | 0.198381373 |
| **HLA-DPA1** | 0.253714871 | 15.00196141 | 27.44461962 | 0.040787282 |
| **HLA-DRB5** | 0.246918215 | 15.21376711 | 31.90958522 | 0.028403624 |
| **HLA-DMA** | 0.241900204 | 14.85010247 | 26.01743205 | 0.070442571 |
| **HLA-DRA** | 0.23293935 | 15.62947066 | 129.9802684 | 0.01357341 |
| **HLA-DRB1** | 0.219527241 | 15.5177292 | 77.08311423 | 0.026867656 |
| **TNFRSF14** | 0.21396525 | 14.39353992 | 12.76512095 | 0.211493261 |
| **NECTIN2** | 0.204164276 | 15.02351804 | 16.39913399 | 0.099511132 |
| **HLA-DQB1** | 0.175215928 | 14.86193216 | 6.207310449 | 0.193163993 |
| **HLA-DQA1** | 0.160761893 | 14.67066617 | 4.896192536 | 0.279517831 |
| **HLA-E** | 0.159366426 | 15.62122069 | 17.04731033 | 0.097070841 |
| **CD226** | 0.128573681 | 13.39958597 | 1.335511374 | 0.743779647 |
| **CD244** | 0.128009997 | 13.58781455 | 0.807718629 | 0.690510312 |
| **HLA-DMB** | 0.074575432 | 14.44198194 | 0.811085634 | 0.662804025 |
| **ICOSLG** | 0.070573246 | 13.38218775 | 0.193611717 | 0.860307268 |
| **DLX3** | 0.053099007 | 14.43848239 | 0.136320745 | 0.757631947 |
| **CD40** | 0.052494061 | 14.66602799 | 0.642544994 | 0.730061931 |
| **HLA-C** | 0.051743367 | 15.71100613 | 1.921914135 | 0.583903729 |
| **HLA-A** | 0.050307858 | 15.72672046 | 1.562412882 | 0.592086781 |
| **CD160** | 0.049485646 | 13.34470404 | 0.300046445 | 0.901961333 |
| **LGALS9** | 0.046067926 | 14.47870227 | 0.402220995 | 0.784483715 |
| **CD96** | 0.039210875 | 13.81922977 | 0.095123262 | 0.881256778 |
| **CD44** | 0.03324297 | 15.3751101 | 0.320786721 | 0.761054496 |
| **HLA-B** | 0.032715361 | 15.78803937 | 0.754827486 | 0.721755438 |
| **CD47** | 0.015407329 | 14.95729551 | 0.07744903 | 0.907505155 |
| **TPH2** | -0.004974329 | 13.28025175 | 0.065659923 | 0.975675591 |
| **BTN2A1** | -0.005044542 | 14.43254089 | 0.006475083 | 0.976947389 |
| **NRP1** | -0.006757175 | 14.47682841 | 0.007398639 | 0.968356518 |
| **PDCD1** | -0.009172923 | 13.86423832 | 0.004067329 | 0.971327192 |
| **BTLA** | -0.024974053 | 13.42209048 | 0.029618301 | 0.94942475 |
| **BTNL3** | -0.032757082 | 13.29041891 | 0.365445214 | 0.905202254 |
| **CD28** | -0.03822209 | 13.6279554 | 0.065574122 | 0.904145142 |
| **CD209** | -0.050145798 | 13.96928958 | 0.105933287 | 0.832505485 |
| **TMIGD2** | -0.054126956 | 13.54516631 | 0.102877837 | 0.876050755 |
| **TNFSF14** | -0.057180121 | 13.56839014 | 0.126700981 | 0.865925537 |
| **BTN2A2** | -0.057729372 | 14.01431175 | 0.561894991 | 0.801634536 |
| **SIRPA** | -0.092522414 | 14.92136821 | 2.091991671 | 0.501792076 |
| **ADORA2A** | -0.101124574 | 13.31463832 | 2.398395027 | 0.785300193 |
| **KIR3DL1** | -0.109529958 | 13.2967642 | 2.536666702 | 0.720942091 |
| **KIR2DL1** | -0.118613741 | 13.30659656 | 1.400071416 | 0.733698586 |
| **CD27** | -0.11955347 | 14.17149755 | 0.852908569 | 0.567141441 |
| **KIR3DL3** | -0.128325112 | 13.28607505 | 7.464879768 | 0.539026652 |
| **HCRTR2** | -0.134110576 | 13.29583434 | 2.457716429 | 0.652720829 |
| **PVR** | -0.158151084 | 14.83229451 | 5.358083005 | 0.277653703 |
| **BTN3A1** | -0.189024248 | 14.45966274 | 9.780124849 | 0.28850231 |
| **KIR2DL3** | -0.190653847 | 13.29917323 | 8.081027104 | 0.538505348 |
| **KIR3DL2** | -0.195604183 | 13.31204209 | 3.419675185 | 0.588186516 |
| **PLEKHG5** | -0.19822539 | 14.61832616 | 5.526902925 | 0.225887668 |
| **LAIR1** | -0.198589545 | 14.0010641 | 2.740346481 | 0.404391727 |
| **IDO2** | -0.206444281 | 13.32097399 | 4.550771421 | 0.588448695 |
| **TNFSF18** | -0.207443246 | 13.69904515 | 0.581438201 | 0.496720632 |
| **HLA-F** | -0.209600659 | 14.98683032 | 15.29597461 | 0.124899509 |
| **HLA-DOB** | -0.242409681 | 14.03211163 | 5.457488131 | 0.302048066 |
| **TNFRSF12A** | -0.290201802 | 15.19080893 | 16.94808555 | 0.021585716 |
| **CD274** | -0.295761053 | 14.25291993 | 5.052065557 | 0.149490038 |
| **HAVCR2** | -0.305381728 | 14.21028888 | 11.06762146 | 0.148025894 |
| **CD86** | -0.307315923 | 14.25798778 | 16.08554292 | 0.133868276 |
| **TNFRSF25** | -0.45264729 | 14.32128154 | 14.57257777 | 0.025668081 |
| **TNFRSF18** | -0.463462871 | 14.62536244 | 12.79115304 | 0.006933919 |
| **CD276** | -0.48970812 | 14.9839056 | 46.16662864 | 0.00069798 |
| **HLA-G** | -0.54047383 | 14.26342736 | 13.01789186 | 0.011402662 |
| **TNFSF9** | -0.554073665 | 14.37842459 | 16.6017347 | 0.005609636 |
| **TNFRSF8** | -0.622305159 | 13.66607574 | 11.22974083 | 0.066009102 |
| **KIR2DS4** | -0.666243136 | 13.34705176 | 13.62934565 | 0.102777287 |
| **ICOS** | -0.704105265 | 13.87597651 | 22.61310348 | 0.014248556 |
| **TIGIT** | -0.707869984 | 13.837527 | 21.56702836 | 0.016911185 |
| **TNFRSF4** | -0.767824706 | 14.11456768 | 43.10227694 | 0.001732525 |
| **LAG3** | -0.786404164 | 14.12650677 | 25.40545992 | 0.001271121 |
| **KIR2DL4** | -0.858938127 | 13.58085509 | 15.14545296 | 0.02443514 |
| **PDCD1LG2** | -0.905696349 | 14.16475519 | 40.52949917 | 0.000199438 |
| **CTLA4** | -0.916611857 | 13.99232757 | 43.45323362 | 0.000821686 |
| **TNFRSF9** | -0.975221376 | 13.76002659 | 40.59129762 | 0.003244263 |
| **TDO2** | -0.995818576 | 13.81748608 | 18.7082665 | 0.001691883 |
| **IDO1** | -1.009319134 | 14.47397869 | 27.48667413 | 1.06E-06 |
| **CD80** | -1.104910042 | 13.58711562 | 52.61175138 | 0.005095661 |
| **TNFSF4** | -1.331389487 | 13.78870431 | 43.90833828 | 0.000110226 |
| **CD70** | -1.421677074 | 13.96870612 | 34.59828716 | 3.27E-06 |

**Table S4.** The differential expression information of differentially expressed genes within 88 ICGs in OSCC samples compared with healthy control samples.

| **genes** | **expression pattern** | **logFC** | **logCPM** | **F** | **PValue** |
| --- | --- | --- | --- | --- | --- |
| VTCN1 | up-regulated DEG | 1.204670031 | 13.74045104 | 24.38987235 | 1.20E-06 |
| CEACAM1 | up-regulated DEG | 1.187418948 | 14.28389901 | 102.4626474 | 8.83E-15 |
| TNFSF15 | up-regulated DEG | 1.046883771 | 13.60919238 | 55.11694022 | 9.87E-05 |
| CD200R1 | up-regulated DEG | 0.952862188 | 13.5390411 | 54.37996505 | 0.001273126 |
| CD40LG | up-regulated DEG | 0.940153382 | 13.4754551 | 37.39746348 | 0.003326497 |
| VSIR | up-regulated DEG | 0.283387027 | 14.83205828 | 26.6519737 | 0.034237965 |
| HLA-DPB1 | up-regulated DEG | 0.271958117 | 15.17744142 | 66.66324327 | 0.017027459 |
| HLA-DPA1 | up-regulated DEG | 0.253714871 | 15.00196141 | 27.44461962 | 0.040787282 |
| HLA-DRB5 | up-regulated DEG | 0.246918215 | 15.21376711 | 31.90958522 | 0.028403624 |
| HLA-DRA | up-regulated DEG | 0.23293935 | 15.62947066 | 129.9802684 | 0.01357341 |
| HLA-DRB1 | up-regulated DEG | 0.219527241 | 15.5177292 | 77.08311423 | 0.026867656 |
| TNFRSF12A | down-regulated DEG | -0.290201802 | 15.19080893 | 16.94808555 | 0.021585716 |
| TNFRSF25 | down-regulated DEG | -0.45264729 | 14.32128154 | 14.57257777 | 0.025668081 |
| TNFRSF18 | down-regulated DEG | -0.463462871 | 14.62536244 | 12.79115304 | 0.006933919 |
| CD276 | down-regulated DEG | -0.48970812 | 14.9839056 | 46.16662864 | 0.00069798 |
| HLA-G | down-regulated DEG | -0.54047383 | 14.26342736 | 13.01789186 | 0.011402662 |
| TNFSF9 | down-regulated DEG | -0.554073665 | 14.37842459 | 16.6017347 | 0.005609636 |
| ICOS | down-regulated DEG | -0.704105265 | 13.87597651 | 22.61310348 | 0.014248556 |
| TIGIT | down-regulated DEG | -0.707869984 | 13.837527 | 21.56702836 | 0.016911185 |
| TNFRSF4 | down-regulated DEG | -0.767824706 | 14.11456768 | 43.10227694 | 0.001732525 |
| LAG3 | down-regulated DEG | -0.786404164 | 14.12650677 | 25.40545992 | 0.001271121 |
| KIR2DL4 | down-regulated DEG | -0.858938127 | 13.58085509 | 15.14545296 | 0.02443514 |
| PDCD1LG2 | down-regulated DEG | -0.905696349 | 14.16475519 | 40.52949917 | 0.000199438 |
| CTLA4 | down-regulated DEG | -0.916611857 | 13.99232757 | 43.45323362 | 0.000821686 |
| TNFRSF9 | down-regulated DEG | -0.975221376 | 13.76002659 | 40.59129762 | 0.003244263 |
| TDO2 | down-regulated DEG | -0.995818576 | 13.81748608 | 18.7082665 | 0.001691883 |
| IDO1 | down-regulated DEG | -1.009319134 | 14.47397869 | 27.48667413 | 1.06E-06 |
| CD80 | down-regulated DEG | -1.104910042 | 13.58711562 | 52.61175138 | 0.005095661 |
| TNFSF4 | down-regulated DEG | -1.331389487 | 13.78870431 | 43.90833828 | 0.000110226 |
| CD70 | down-regulated DEG | -1.421677074 | 13.96870612 | 34.59828716 | 3.27E-06 |

**Table S5.** The hazard ratio (HR)-related parameters (i.e., HR, HR with lower/higher 95% confidence index) and p values of eight CD274-positively correlated ICGs (CTLA4, ICOS, TNFRSF4, CD27, BTLA, ADORA2A, CD40LG, CD28).

| Gene | HR | HR.95L | HR.95H | Pvalue |
| --- | --- | --- | --- | --- |
| CTLA4 | 0.718789 | 0.574955 | 0.898604 | 0.00375 |
| ICOS | 0.702284 | 0.546962 | 0.901713 | 0.005585 |
| TNFRSF4 | 0.721183 | 0.572246 | 0.908884 | 0.005615 |
| CD27 | 0.815736 | 0.69291 | 0.960334 | 0.014443 |
| BTLA | 0.356026 | 0.151753 | 0.835265 | 0.017611 |
| ADORA2A | 0.012488 | 0.00029 | 0.53712 | 0.022382 |
| CD40LG | 0.498682 | 0.270096 | 0.920722 | 0.02615 |
| CD28 | 0.639389 | 0.426963 | 0.957504 | 0.029951 |

**Table S6.** The correlation between eight CD274-positively correlated ICGs (CTLA4, ICOS, TNFRSF4, CD27, BTLA, ADORA2A, CD40LG, CD28) and tumour mutation burden.

| CD274-related ICGs | cor | pValue | sig |
| --- | --- | --- | --- |
| CTLA4 | -0.14706 | 0.00842 | ** |
| ICOS | -0.17017 | 0.002254 | ** |
| TNFRSF4 | -0.20036 | 0.00031 | *** |
| CD27 | -0.18719 | 0.000765 | *** |
| BTLA | -0.19895 | 0.000342 | *** |
| ADORA2A | -0.10841 | 0.052694 |  |
| CD40LG | -0.18912 | 0.000672 | *** |
| CD28 | -0.2258 | 4.58E-05 | *** |

**Table S7.** The relationship between overall survival and CD274-related ICGs (CD274 and its eight positively correlated ICGs), based on the TCGA-OSCC data analysis.

| **ICGs** | **Favorable survival** | **Poor survival** | **P values** | **Statistical significance** |
| --- | --- | --- | --- | --- |
| **CD274** | CD274_low | CD274_high | p = 0.57 | no |
| **BTLA** | BTLA_low | BTLA_high | p = 0.0069 | yes |
| **CD27** | CD27_low | CD27_high | p = 0.0056 | yes |
| **CTLA4** | CTLA4_low | CTLA4_high | p = 0.0062 | yes |
| **CD28** | CD28_low | CD28_high | p = 0.049 | yes |
| **CD40LG** | CD40LG_low | CD40LG_high | p = 0.046 | yes |
| **ICOS** | ICOS_low | ICOS_high | p = 0.041 | yes |
| **TNFRSF4** | TNFRSF4_low | TNFRSF4_high | p = 0.015 | yes |
| **ADORA2A** | ADORA2A_low | ADORA2A_high | p = 0.22 | no |

**Table S8.** The relationship between overall survival and CD274-related ICGs genes pairs subtypes, based on the TCGA-OSCC data analysis. These subtypes consisted of two categories: (i) CD274 and its positively correlated ICGs; (ii) CD8A and CD274-positively correlated ICGs.

| **ICGs combination pairs** | **Best survival** | **Worst survival** | **P values** | **Statistical significance** |
| --- | --- | --- | --- | --- |
| **Combination pairs consisting of CD274 and its positively correlated ICGs:** | | | | |
| **CD274-BTLA** | CD274_low/BTLA_low | CD274_high/BTLA_high | p = 0.019 | yes |
| **CD274-CD27** | CD274_low/CD27_low | CD274_high/CD27_high | p = 0.015 | yes |
| **CD274-CTLA4** | CD274_low/CTLA4_low | CD274_high/CTLA4_high | p = 0.0052 | yes |
| **CD274-CD28** | CD274_low/CD28_low | CD274_high/CD28_high | p = 0.13 | no |
| **CD274-CD40LG** | CD274_low/CD40LG_low | CD274_high/CD40LG_high | p = 0.086 | no |
| **CD274-ICOS** | CD274_low/ICOS_low | CD274_high/ICOS_high | p = 0.051 | no |
| **CD274-TNFRSF4** | CD274_low/TNFRSF4_low | CD274_high/TNFRSF4_high | p = 0.052 | no |
| **CD274-ADORA2A** | CD274_low/ADORA2A_low | CD274_high/ADORA2A_high | p = 0.51 | no |
| **Combination pairs consisting of CD8A and CD274-positively correlated ICGs:** | | | | |
| **CD8A-CD274** | CD8A_low/CD274_low | CD8A_high/CD274_high | p = 0.46 | no |
| **CD8A-BTLA** | CD8A_high/BTLA_low | CD8A_high/BTLA_high | p = 0.019 | yes |
| **CD8A-CD27** | CD8A_high/CD27_low | CD8A_high/CD27_high | p = 0.025 | yes |
| **CD8A-CTLA4** | CD8A_high/CTLA4_low | CD8A_high/CTLA4_high | p = 0.032 | yes |
| **CD8A-CD28** | CD8A_high/CD28_low | CD8A_high/CD28_high | p = 0.27 | no |
| **CD8A-CD40LG** | CD8A_high/CD40LG_low | CD8A_high/CD40LG_high | p = 0.26 | no |
| **CD8A-ICOS** | CD8A_high/ICOS_low | CD8A_high/ICOS_high | p = 0.22 | no |
| **CD8A-TNFRSF4** | CD8A_high/TNFRSF4_low | CD8A_high/TNFRSF4_high | p = 0.031 | yes |
| **CD8A-ADORA2A** | CD8A_high/ADORA2A_low | CD8A_high/ADORA2A_high | p = 0.42 | no |

**Fig S1.** The Kaplan-Meier plots showing the significant relationship between 5-year overall survival and gene pairs subtypes consisted of CD274-related genes, based on the data analysis of GSE41613 dataset. (A) The KM plots showing the prognostic values of subtypes consisted of CD274 and its positively correlated ICGs (BTLA, CD27, and CTLA4). (B) The KM plots showing the prognostic values of CD8A and three CD274-positively correlated ICGs (BTLA, CD27, and CTLA4).

**
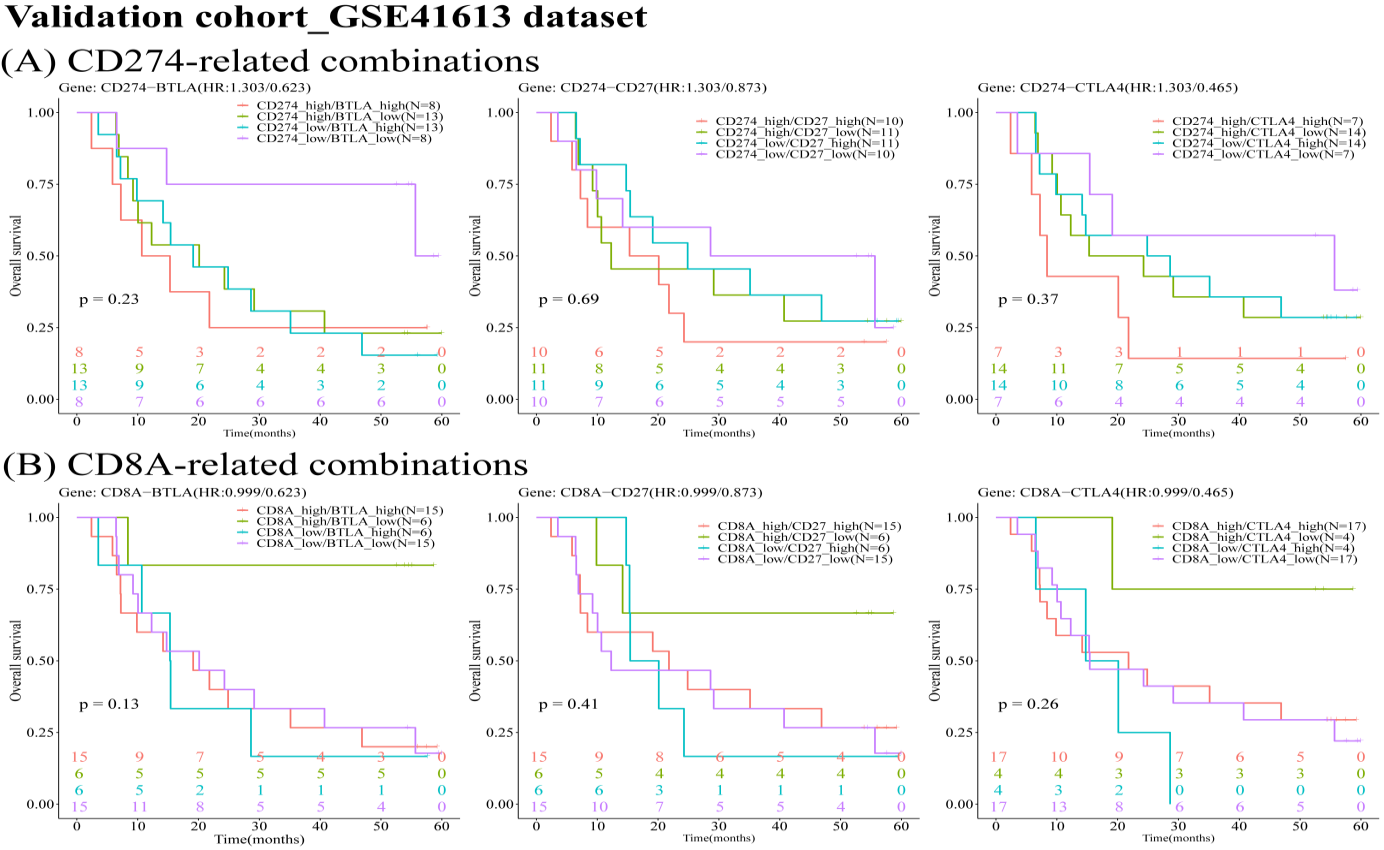
**

**Fig S2.** The Kaplan-Meier plots showing the significant relationship between 5-year overall survival and gene pairs subtypes consisted of CD274-related genes, based on the data analysis of GSE42743 dataset. (A) The KM plots showing the prognostic values of subtypes consisted of CD274 and its positively correlated ICGs (BTLA, CD27, and CTLA4). (B) The KM plots showing the prognostic values of CD8A and three CD274-positively correlated ICGs (BTLA, CD27, and CTLA4).

**
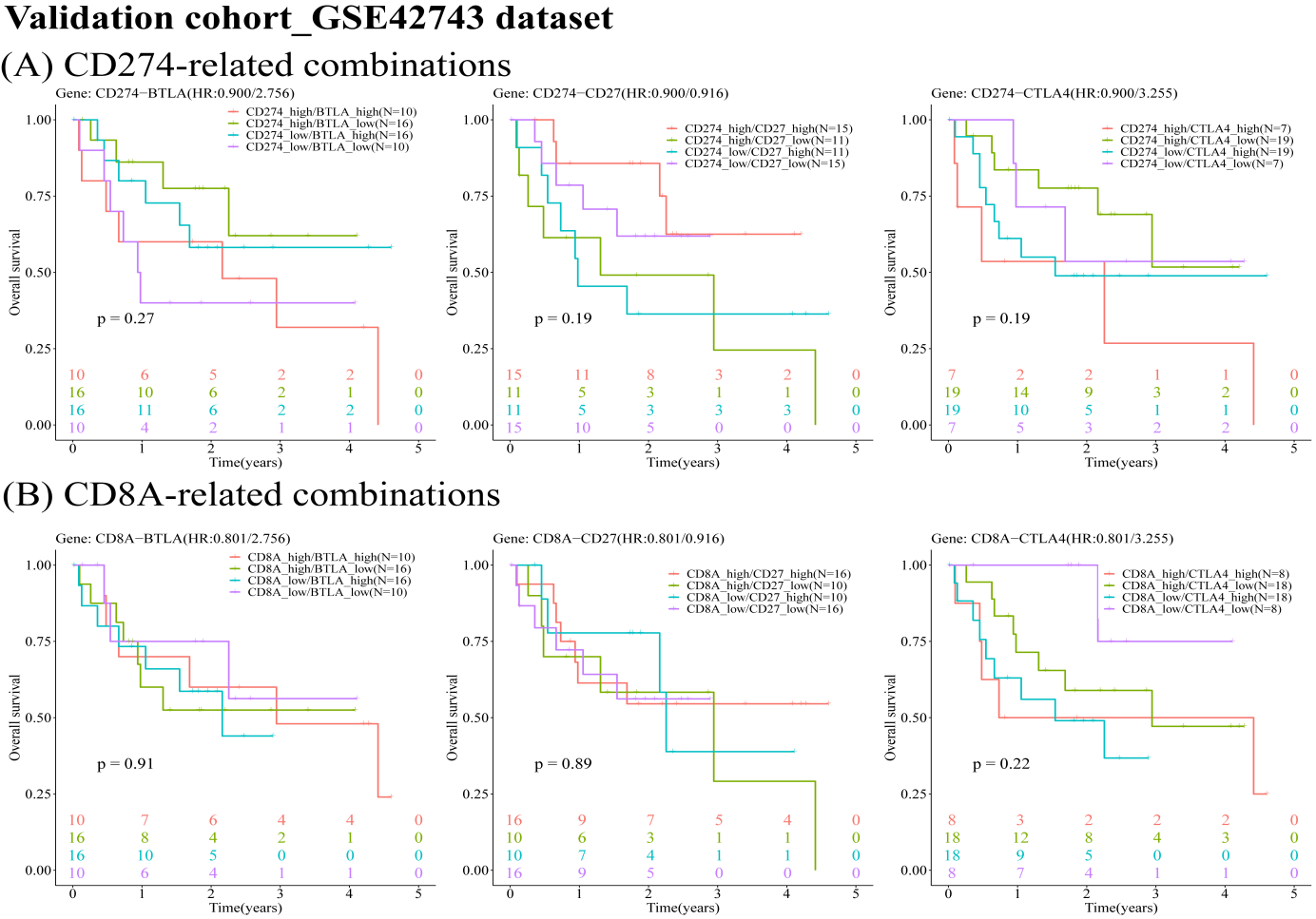
**

**Fig S3.** The Kaplan-Meier plots showing the significant relationship between 5-year overall survival and gene pairs subtypes consisted of CD274-related genes, based on the data analysis of GSE75538 dataset. (A) The KM plots showing the prognostic values of subtypes consisted of CD274 and its positively correlated ICGs (BTLA, CD27, and CTLA4). (B) The KM plots showing the prognostic values of CD8A and three CD274-positively correlated ICGs (BTLA, CD27, and CTLA4).

**
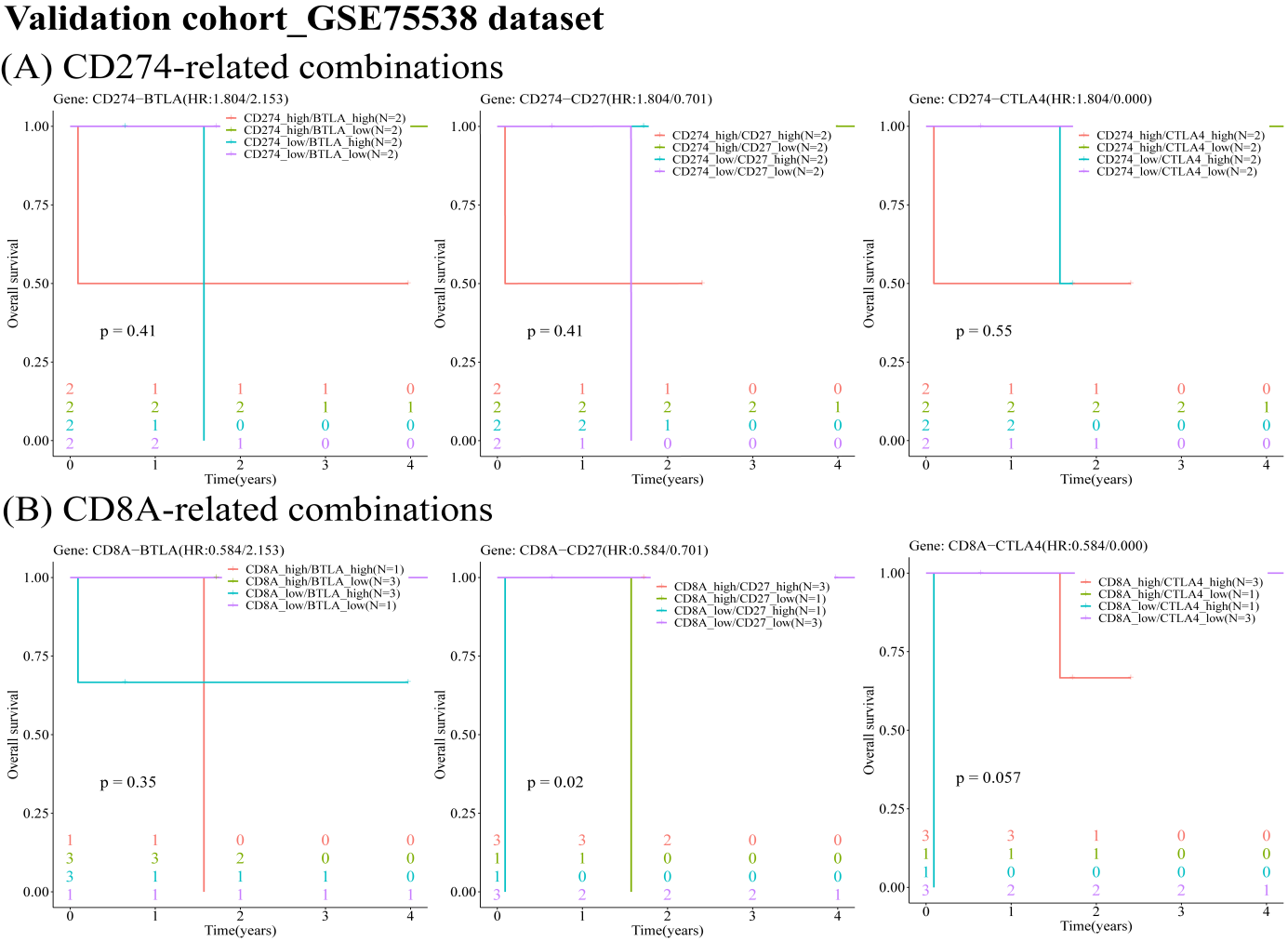
**

**Fig S4.** The Kaplan-Meier plots showing the significant relationship between 5-year overall survival and gene pairs subtypes consisted of CD274-related genes, based on the data analysis of GSE85446 dataset. (A) The KM plots showing the prognostic values of subtypes consisted of CD274 and its positively correlated ICGs (BTLA, CD27, and CTLA4). (B) The KM plots showing the prognostic values of CD8A and three CD274-positively correlated ICGs (BTLA, CD27, and CTLA4).

**
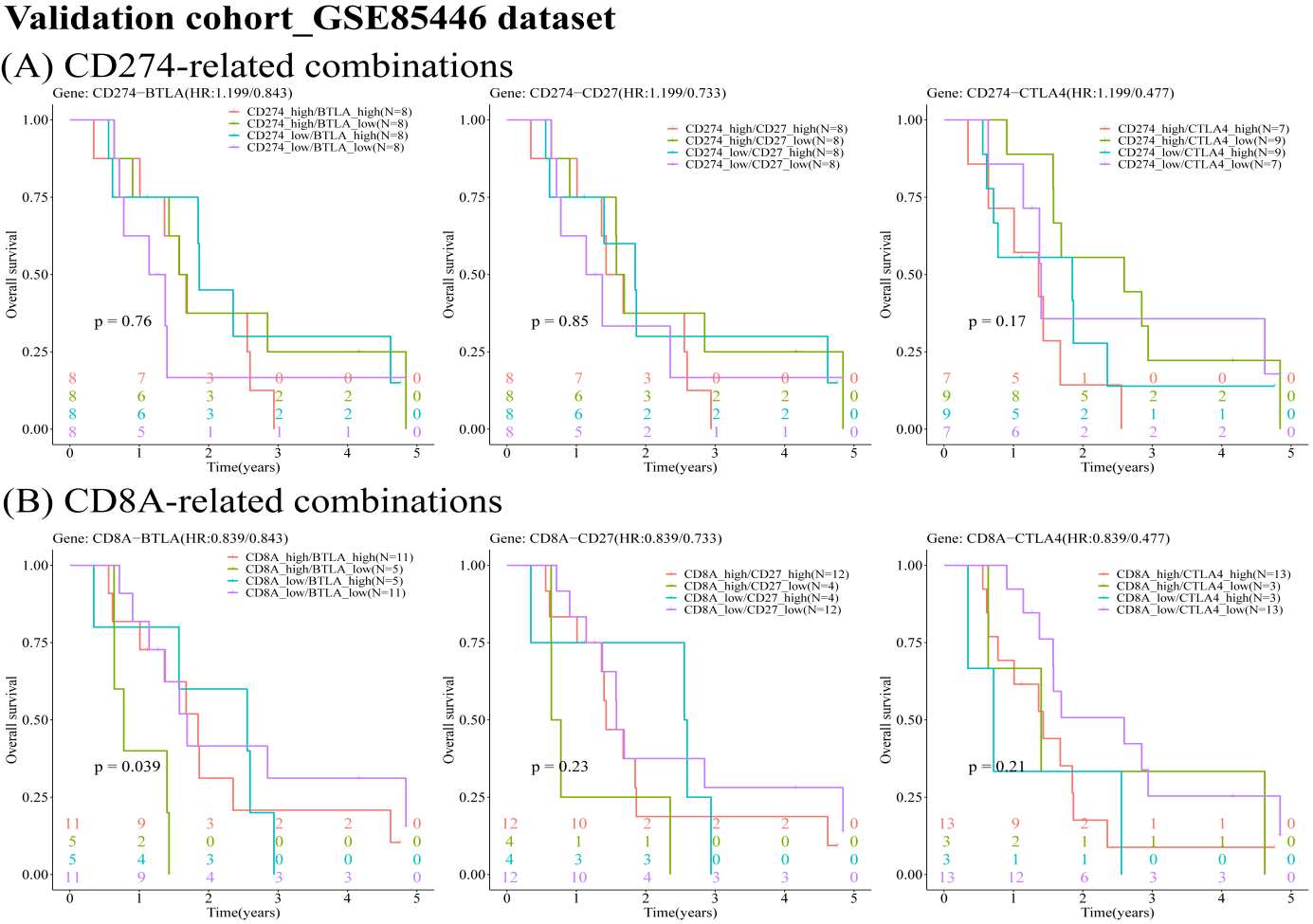
**
